# Conquering the Sahara and Arabian deserts: systematics and biogeography of *Stenodactylus* geckos (Reptilia: Gekkonidae)

**DOI:** 10.1186/1471-2148-12-258

**Published:** 2012-12-31

**Authors:** Margarita Metallinou, Edwin Nicholas Arnold, Pierre-André Crochet, Philippe Geniez, José Carlos Brito, Petros Lymberakis, Sherif Baha El Din, Roberto Sindaco, Michael Robinson, Salvador Carranza

**Affiliations:** 1 Institute of Evolutionary Biology (CSIC-UPF), Passeig Marítim de la Barceloneta 37-49, 08003, Barcelona, Spain; 2 The Natural History Museum, Cromwell Road, SW7 5BD, London, UK; 3 CNRS-UMR 5175 Centre d'Ecologie Fontionnelle et Evolutive, 1919 Route de Mende, 34293, Montpellier cedex 5, France; 4 EPHE-UMR, Centre d'Ecologie Fontionnelle et Evolutive, 1919 Route de Mende, 34293, Montpellier cedex 5, France; 5 CIBIO/InBIO, Centro de Investigação em Biodiversidade e Recursos Genéticos da Universidade do Porto, Instituto de Ciências Agrárias de Vairão, R. Padre Armando Quintas 4485-661, Vairão, Portugal; 6 Natural History Museum of Crete, University of Crete, Knosou Av, P.O. Box 2208, 71409, Heraklion, Greece; 7 Nature Conservation Sector, Egyptian Environmental Affairs Agency, 3 Abdalla El Katib, Apt. 3, Cairo, Dokki, Egypt; 8 Museo Civico de Storia Naturale, via San Francesco di Sales 188, I-10022, Carmagnola, Italy; 9 Sultan Qaboos University, Department of Biology, College of Science, Al-Khod, P.O. Box 36, Muscat, Sultanate of Oman

**Keywords:** *Stenodactylus*, Gekkonidae, Arabia, North Africa, Phylogeny, Biogeography, Desert, Red Sea

## Abstract

**Background:**

The evolutionary history of the biota of North Africa and Arabia is inextricably tied to the complex geological and climatic evolution that gave rise to the prevalent deserts of these areas. Reptiles constitute an exemplary group in the study of the arid environments with numerous well-adapted members, while recent studies using reptiles as models have unveiled interesting biogeographical and diversification patterns. In this study, we include 207 specimens belonging to all 12 recognized species of the genus *Stenodactylus*. Molecular phylogenies inferred using two mitochondrial (12S rRNA and 16S rRNA) and two nuclear (c-*mos* and *RAG*-2) markers are employed to obtain a robust time-calibrated phylogeny, as the base to investigate the inter- and intraspecific relationships and to elucidate the biogeographical history of *Stenodactylus*, a genus with a large distribution range including the arid and hyper-arid areas of North Africa and Arabia.

**Results:**

The phylogenetic analyses of molecular data reveal the existence of three major clades within the genus *Stenodactylus*, which is supported by previous studies based on morphology. Estimated divergence times between clades and sub-clades are shown to correlate with major geological events of the region, the most important of which is the opening of the Red Sea, while climatic instability in the Miocene is hypothesized to have triggered diversification. High genetic variability is observed in some species, suggesting the existence of some undescribed species. The *S. petrii* - *S. stenurus* species complex is in need of a thorough taxonomic revision. New data is presented on the distribution of the sister species *S. sthenodactylus* and *S. mauritanicus*.

**Conclusions:**

The phylogenetic hypothesis for the genus *Stenodactylus* presented in this work permits the reconstruction of the biogeographical history of these common desert dwellers and confirms the importance of the opening of the Red Sea and the climatic oscillations of the Miocene as major factors in the diversification of the biota of North Africa and Arabia. Moreover, this study traces the evolution of this widely distributed and highly specialized group, investigates the patterns of its high intraspecific diversity and elucidates its systematics.

## Background

North Africa and Arabia are home to a unique fauna and flora that has been shaped by the combination of several factors including the harsh climatic conditions of the Sahara and Arabian deserts, the episodic appearance of humid cycles, and by the complex geological evolution of the area [1-9]. One of the most important geological phenomena of the entire Cenozoic that occurred in this area was the break-up of the Arabian plate from Africa. Tectonic activity started approximately 30 Ma ago at the central Gulf of Aden with the formation of a rift basin in the Eritrean Red Sea and initial rifting at the Afar zone. A second phase of volcanism occurred 24 Ma ago, causing extension and rifting throughout the entire Red Sea, from Yemen to Egypt, as well as uplifting of the newly-formed continental shoulders [1]. Nevertheless, fluctuations of the sea level during the Miocene permitted the formation of transient land connections [1,10] that were subsequently lost [11].

The establishment of the Afro-Arabia - Eurasia land bridge (*Gomphotherium* bridge) was another crucial event with major biogeographical implications [12-14]. Following the opening of the Gulf of Aden and the Red Sea and with the counterclockwise rotation of the Arabian plate, a first connection was presumably formed between the latter and the Anatolian plate, and subsequently with Eurasia. Although the connection between the Mediterranean Sea and the Indian Ocean is hypothesized to have been re-established in the Upper Middle Miocene, around 15 Ma ago, it is believed that posterior to this date the land bridge has been continuously present [15]. Important faunal and floral exchanges have been attributed to the establishment of this connection ([12-14] and references therein).

Although the origin of the Sahara and Arabian deserts is still hotly debated [16-19], it is generally accepted that climatic development in the late Miocene, as a result of major growth of the East Antarctic Ice Sheet and polar cooling, lead to an increase in aridification of mid-latitude continental regions [4] and that this had a profound effect on the diversification of faunas [20-22].

Reptiles are among the commonest inhabitants of arid areas and have long been used in biogeographic, ecological and evolutionary studies [23], constituting thus excellent models to investigate how diversity is originated and maintained. Several cases of faunal exchanges in both directions between North Africa and Arabia have been described (e.g. [2,13,24]) showing that there is not a single pattern, but rather different hypotheses including both vicariance and dispersal, heavily dependent on the estimated timeframe of the events. Moreover, several studies have shown that climatic changes towards aridity and contraction/expansion of the Sahara and Arabian deserts have played a decisive role in reptile species diversification [25-29].

**Figure 1:**
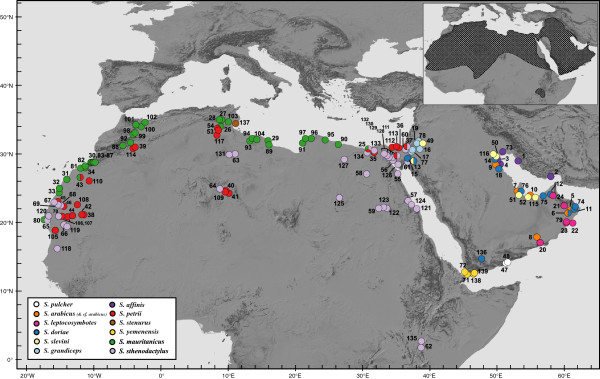
**Sampling localities of the *Stenodactylus *specimens used in this study.** Colors and locality numbers refer to specimens in Figures 2 and 3 (see also Additional file 1: Table S1). The global distribution of the genus is seen on the upper right (data from Sindaco and Jeremcenko, 2008).

Gekkonid lizards of the genus *Stenodactylus* Fitzinger, 1826 [30] are one of the most characteristic and abundant elements of the fauna of the arid and hyper-arid regions of Arabia and North Africa [31]. The genus comprises twelve species that are distributed in a more or less continuous range across northern Africa and Arabia, with an apparently isolated population in northern Kenya and extending around the Arabian Gulf to coastal southwestern Iran ([32,33]; see Figure 1). Up to three species may occur at a single locality and, where such sympatry exists, resource partitioning is largely achieved by microhabitat segregation, with species occupying different soil types [34]. Gravel plains, hard sand and aeolian soft sand all have their characteristic species that show specialized morphological adaptations. These include the presence of depressed and fringed toes, which increase the surface area and improve grip in the aeolian sand dune specialists *Stenodactylus doriae* (Blanford, 1874 [35])*, S. petrii* Anderson, 1896 [36] and *S. arabicus* (Haas, 1957 [37]). Extensive webbing is also observed between the fingers for efficient sand burrowing in *S. arabicus*[31,32,38]. When two species are regularly found on the same substrate, they greatly differ in size and there are corresponding differences in the size of prey taken [32].

Morphologically, *Stenodactylus* is fairly homogeneous and all species exhibit phalangeal reduction that produces a formula of 2.3.3.4.3 on both fore and hind limbs and are also characterized by a very high scleral ossicle number (20–28) [31,39]. A morphology-based phylogenetic hypothesis has been proposed by Arnold (1980) [31]. Although these two characters are also present in *Pseudoceramodactylus khobarensis* Haas, 1957 [37], which was widely accepted as a *Stenodactylus* member [31,39], a recent phylogenetic study by Fujita and Papenfuss (2011) [40] including specimens of the former and six out of the twelve species of the genus *Stenodactylus* proposed the resurrection of the genus *Pseudoceramodactylus*. This was done in order to deal with the resulting paraphyly of *Stenodactylus*, caused by the branching of two representatives of the genus *Tropiocolotes* between *P. khobarensis* and the six *Stenodactylus* included in their analyses*.* Their molecular analyses also uncovered high levels of genetic divergence between the different *Stenodactylus* species. Genetic variability within some of the species, like *S. arabicus* and *S. doriae*, was also high and this could be linked to biogeographic discontinuities among some of the hyper-arid areas in Arabia.

Although *Stenodactylus* includes a relatively low number of species compared to other gecko groups in these areas, such as *Pristurus*, *Tarentola* or *Hemidactylus*[26,41-46], its relatively high level of resource partitioning and habitat specialization has allowed the different species to successfully colonize almost all available habitats in the arid and hyper-arid regions of North Africa and Arabia. It constitutes, therefore, a very interesting, but still poorly studied, genus that makes an excellent model for the study of desert biodiversity and biogeography. The main objectives of the present work are: (1) to provide for the first time a complete phylogeny of the genus *Stenodactylus* and evaluate its concordance with previous molecular and morphology-based studies; (2) to investigate the biogeographical and diversification patterns of *Stenodactylus*; and (3) to explore the interspecific relationships, the patterns of intraspecific diversity and the possible presence of unrecognized divergent lineages in *Stenodactylus*.

## Methods

### Taxon sampling, DNA extraction and sequencing

A total of 207 individuals of *Stenodactylus* representing all twelve currently recognized species were included in the present study. Whenever possible, we tried to include multiple populations for each species in order to assess intraspecific variation; sampling was especially intense in the three African species with very large distribution ranges. In addition, three *Pseudoceramodactylus khobarensis* and eight individuals representing six species of the genus *Tropiocolotes* were included in an attempt to further test the relationship between *Stenodactylus, Pseudoceramodactylus* and *Tropiocolotes*. Four additional specimens from other closely related genera [47-49] were used as outgroups and sixteen specimens, from several genera, were added in order to estimate divergence times (see below). Additional file 1: Table S1 lists all 238 samples used in the present work with their extraction codes, voucher references, localities and GenBank accession numbers (KC190516-KC191151).

Genomic DNA was extracted from ethanol-preserved tissue samples using the DNeasy Blood & Tissue Kit (Qiagen, Valencia, CA, USA). All 222 specimens included in the phylogenetic analyses were sequenced for two mitochondrial gene fragments: 378–388 base pairs (bp) of 12S rRNA (12S) and 498–536 bp of the 16S rRNA (16S). A subset of 106 specimens, including representatives from all independent lineages recovered by the analysis of these two fragments, was also sequenced for two nuclear markers: 660 bp of the oocyte maturation factor MOS (c-*mos*), and 410 bp of the recombination activating gene 2 (*RAG*-2). Primers used for the amplification and sequencing of the 12S, 16S, c-*mos* and *RAG*-2 gene fragments as well as PCR conditions applied in the present work are listed in detail in Table 1. All amplified fragments were sequenced for both strands. Contigs were assembled in Geneious v.5.3 [50].

**Table 1:** Primers used in this study

**Gene fragment**	**Primer name**	**Or.**^**1**^	**Sequence (5′- 3′)**	**Reference**	**PCR conditions**
**12S**	12Sa	F	AAACTGGGATTAGATACCCCACTAT	Kocher et al. (1989)	94º (5'); 94º (45"), 51º (45"), 72º (80") × 35; 72 (5')
	12Sb	R	GAGGGTGACGGGCGGTGTGT	Kocher et al. (1989)	
	L1.STENO	F	GGATTAGATACCCCACTATGC	This study	94º (5'); 94º (45"), 52º (45")’, 72º (90") × 35; 72º (5')
	H1.STENO1	R	TGACGGGCGGTGTGTACG	This study	
**16S**	16Sa	F	CGCCTGTTTATCAAAAACAT	Palumbi (1996)	94º (5'); 94º (45"), 51 (45"), 72 (80") × 35; 72º (5')
	16Sb	R	CCGGTCTGAACTCAGATCACGT	Palumbi (1996)	
	16SaST	F	ATCAAAAACATCGCCTTTAGC	This study	94º (5'); 94º (45"), 57º (45"), 72º (70") × 35; 72º (5')
	16SbST	R	CTGAACTCAGATCACGTAGGAC	This study	
**C-*mos***	FUF	F	TTTGGTTCKGTCTACAAGGCTAC	Gamble et al. (2008)	94º (5'); 94º (30"), 55º (45"), 72º (70") × 35; 72º (10')
	FUR	R	AGGGAACATCCAAAGTCTCCAAT	Gamble et al. (2008)	
	G73_STENO	F	GCTGTAAAGCAGGTGAAGAAATGC	This study	94º (5'); 94º (45"), 56º (45"), 72º (80") × 35; 72º (5')
	G74_STENO	R	GAACATCCAAAGTCTCCAATCTTGC	This study	
	G73.5_STENO	F	GCATTTGGACTTAAAACCTG	This study	
	G708	R	GCTACATCAGCTCTCCARCA	Hugall et al. (2008)	
***RAG*-2**	RAG2-PY1-F	F	CCCTGAGTTTGGATGCTGTACTT	Gamble et al. (2008)	94º (5'); 94º (45"), 55º (45"), 72º (70") × 35; 72º (5')
	RAG2-PY1-R	R	AACTGCCTRTTGTCCCCTGGTAT	Gamble et al. (2008)	

List of primers used in the amplification and sequencing of gene fragments, with the corresponding source and PCR conditions.

^1^Orientation.

### Phylogenetic analyses and hypothesis testing

DNA sequences were aligned using the online version of MAFFT v.6 [51] with default parameters (gap opening = 1.53, offset value = 0.0) for the mitochondrial genes and with modified parameters (offset value = 0.1) for the nuclear genes, in which long gaps are not expected. Coding gene fragments (c-*mos* and *RAG*-2) were translated into amino acids and no stop codons were observed. Uncorrected *p*-distances were calculated in MEGA v.5 [52].

Phylogenetic analyses of the combined dataset were done employing maximum likelihood (ML) and Bayesian (BI) methods. Separate ML analyses were also performed on 12S, 16S, c-*mos* and *RAG*-2 to test for conflicting signal among genes. Best-fitting nucleotide substitution models were selected for each partition under the Akaike information criterion [53] using jModelTest v.0.1.1 [54]. The GTR + I + G model was independently estimated for each of the 12S, 16S, *RAG-*2 partitions and the GTR + G model for the c-*mos* partition. Alignment gaps were treated as missing data and the nuclear gene sequences were not phased. *Hemidactylus frenatus* was used for rooting the tree, based on published evidence [47,48].

A Bayesian analysis of the combined dataset was performed in MrBayes 3.1.2 [55,56] with best fitting models applied to each partition (gene) and all parameters unlinked across partitions. Analyses ran for 10^7^ generations, with sampling intervals of 1000 generations, producing 10000 trees. Convergence and appropriate sampling were confirmed examining the standard deviation of the split frequencies between the two simultaneous runs and the Potential Scale Reduction Factor (PSRF) diagnostic. Burn-in was performed discarding the first 2500 trees of each run and a majority-rule consensus tree was generated from the remaining trees. ML analyses were performed in RAxML v.7.0.3 [57]. A GTR + I + G model was used and parameters were estimated independently for each partition. Node support was assessed by bootstrap analysis [58] including 1000 replications.

Haplotype networks were constructed for the two nuclear markers c-*mos* and *RAG*-2. The software PHASE v.2.1.1 [59,60] was used to resolve the haplotypes where more than one heterozygote position was present. Input files were prepared using Seqphase [61]. In order to include as much information as possible for the better resolution of the haplotypes, the alignment of all full-length sequences of each marker was used. Phase probabilities parameter was set at 0.7 and all other settings were set by default. The network of the resulting haplotypes was calculated with TCS v.1.21 [62] applying default settings (probability of parsimony cutoff: 95%).

Topological constraints to test alternative topologies were constructed by hand and compared to the unconstrained (best) tree using the Approximately-Unbiased (AU) [63] and Shimodaira-Hasegawa (SH) [64] tests. Per-site log likelihoods were estimated in RAxML 7.0.3 [57] and *P*-values were calculated using Consel[65]. Tests were also run in a Bayesian framework, where the relative support of competing hypotheses given the data was quantified using the Bayes factor (BF) [66]. Topologies were constrained in analyses run in BEAST v.1.6.1 [67], the marginal likelihood for each topology was estimated using the harmonic mean estimator and the Bayes factors were calculated by taking the ratios, as estimated in Tracer v.1.5 [68].

### Estimation of divergence times

A Bayesian approach was used to estimate divergence times as implemented in the software BEAST v.1.6.1. The dataset comprised sequences from all four partitions (the nuclear genes c-*mos* and *RAG*-2 unphased). An arbitrarily pruned phylogeny was used in order to include only one representative from each species or main lineage uncovered with the concatenated analysis (45 specimens in total; see Additional file 1: Table S1). This method excludes closely related terminal taxa because the Yule tree prior does not include a model of coalescence, which can complicate rate estimation for closely related sequences [69]. Additionally, several taxa belonging to other gecko genera were added for the calibration process (see below).

Two individual runs were performed for 4 × 10^7^ generations with a sampling frequency of 4000 and the results were combined to infer the ultrametric tree after discarding 10% of the samples from each run. Models and prior specifications applied were as follows (otherwise by default): GTR + I + G (12S, 16S), GTR + I (c-*mos*), HKY + I (*RAG*-2); Relaxed Uncorrelated Lognormal Clock (estimate); Yule process of speciation; random starting tree; alpha Uniform (0, 10); yule.birthRate (0, 1000). Parameter values both for clock and substitution models were unlinked across partitions.

Unfortunately, no fossils belonging to *Stenodactylus*, *Pseudoceramodactylus* or *Tropiocolotes* are known, precluding the direct estimation of the time of the cladogenetic events within our study group. Consequently, the estimation was based on well-known calibration points published in recent literature [70,71] related to members of the families Phyllodactylidae and Sphaerodactylidae (see Additional file 1: Table S1). Three fossil and biogeographical calibration points were applied as “soft” priors, in order to account for uncertainty in the date of the corresponding nodes: (1) the minimum age for the divergence between *Euleptes* and its sister clade was set to 22.5 Ma ago using the approximate age of a fossil *Euleptes*[72,73] (Lognormal distribution: median 22.5, 97.5% 36.55); (2) the split between *Teratoscincus scincus* - *Teratoscincus roborowskii* caused by the Tien Shan-Pamir uplift 10 Ma ago [74-76] (Lognormal distribution: median 10.08, 97.5% 12.96); (3) the age of El Hierro island [77] at 1.12 Ma ago, assuming that divergence between *Tarentola boettgeri hierrensis* and *Tarentola boettgeri bischoffi* began soon after its appearance [26,44] (Uniform distribution: lower 1, upper 1.12). In order to cross-check the results, the posterior mean rates of the mitochondrial gene fragments of our analysis were compared to the rates calculated for well-known and well-studied reptile groups from the Canary Islands (the geckos of the genus *Tarentola*, the lacertid lizards of the endemic genus *Gallotia* and the skinks of the genus *Chalcides*), for which robust calibrated phylogenies have been produced in several independent analyses ([26,45,78-80], among others), and evolutionary rates for the 12S gene have been obtained using BEAST [44].

### Ancestral area reconstruction

MacClade v. 4.08 [81] was used to reconstruct the ancestral areas for the *Stenodactylus* species in a parsimony framework, using both delayed transformation (DELTRAN) and accelerated transformation (ACCTRAN). Additionally, in order to incorporate branch-length information, ML was used as implemented in the Mesquite software package [82]. Both Markov k-state 1-parameter and Asymmetrical Markov k-state 2-parameter models were applied and a likelihood ratio test was used to choose the best reconstruction. Two states, Arabia and Africa, were identified in the extant species depending on the present distribution of the species [33] and were used with both methodologies.

## Results

### Phylogenetic analyses and topological tests

**Figure 2:**
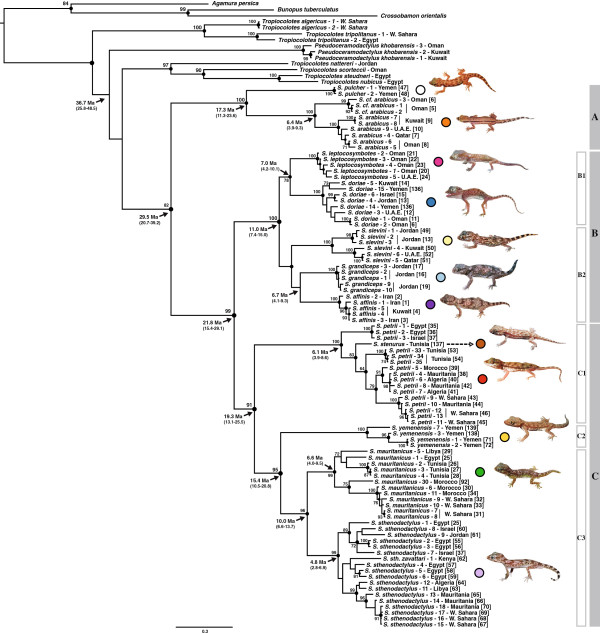
**BI tree of the genus *Stenodactylus *inferred using 12S, 16S mtDNA and c-*mos*, *RAG*-2 nuclear gene fragments.** Black circles on the nodes indicate posterior probability values above 0.95 in the Bayesian Inference analysis. Numbers next to the nodes indicate bootstrap support of the Maximum Likelihood analysis (only values above 70 are shown). Ages of the nodes estimated with BEAST are indicated with an arrow, with the corresponding age range in brackets. The tree was rooted using *Hemidactylus frenatus*. Numbers in square brackets next to specimens code refer to localities in Figure 1. Information on the samples included is shown in Additional file 1: Table S1. Species' pictures were not submitted to precise relative scaling.

**Figure 3:**
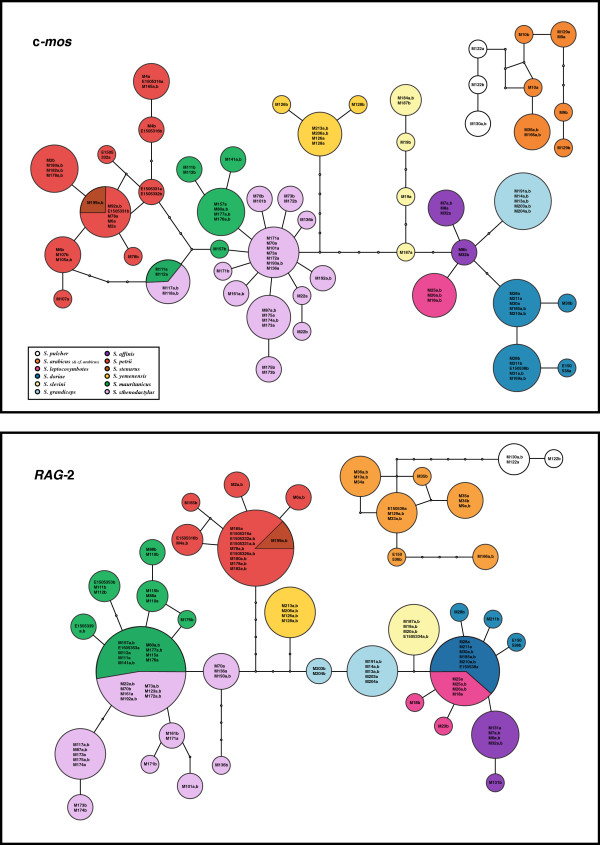
**Haplotype networks of the nuclear markers c-*mos *and *RAG*-2.** Only full-length sequences were used and phase probabilities were set as ≥ 0.7. Information on the samples included is shown in Additional file 1: Table S1.

Two datasets were used to infer the phylogenetic relationships of the genus *Stenodactylus*: a mitochondrial one for building the preliminary phylogeny and analyzing the divergence patters, and a multi-locus one for producing a more robust phylogeny (TreeBASE ID: 13567). The first dataset consisted of an alignment of 974 bp of mitochondrial DNA (415 bp of 12S and 559 bp of 16S, of which 270 in both cases were variable positions) for 222 terminals including 207 *Stenodactylus*. The results of the ML and BI of this dataset were very similar and are summarized in Supplementary Figure 1 (Additional file 2: Figure S1). In order to improve our phylogenetic hypothesis applying a multi-locus approach, a second dataset was assembled with a selection of 106 terminals, including 91 *Stenodactylus* (see Additional file 1: Table S1) for which two extra nuclear genes were sequenced. The aligned dataset consisted of 2092 bp (419 bp of 12S, 560 bp of 16S, 703 bp of c-*mos* and 410 bp of *RAG*-2, of which 262, 269, 109 and 99 positions were variable, respectively). The result of the phylogenetic analyses of the concatenated alignment of four genes is shown in Figure 2. Well-supported relationships in the independent gene trees were congruent among partitions, but at this level not all markers offered sufficient resolution to differentiate particularly between *S. sthenodactylus* and *S. mauritanicus* (data not shown but see below). The networks constructed for the phased haplotypes of the nuclear markers are presented in Figure 3. Not all ambiguities were resolved.

Both ML and Bayesian analyses of the concatenated alignment of four gene fragments (Figure 2) gave almost identical results to the mtDNA tree from the Additional file 2: Figure S1 There is low support over the relationships between the genera *Stenodactylus, Pseudoceramodactylus* and *Tropiocolotes*. According to the results, the North African *T. algericus* and *T. tripolitanus* branch first and *P. khobarensis* is sister to a poorly supported clade formed by two reciprocally monophyletic groups: one including *T. scorteccii, T. steudneri*, *T. nubicus* and the Middle Eastern *T. nattereri* and the other one including all 12 species of the genus *Stenodactylus*. In order to further investigate these relationships, three topological tests were carried out: (1) *Stenodactylus* + *Pseudoceramodactylus* were forced monophyletic; (2) *Tropiocolotes* was forced monophyletic; and (3) *Stenodactylus* + *Pseudoceramodactylus* were forced monophyletic and *Tropiocolotes* was forced monophyletic on the same constraint tree. The resulting constrained topologies were compared to our optimal topology from Figure 2 under both ML and Bayesian frameworks (see Table 2). The results of the topological tests indicate that our dataset cannot reject the alternative hypothesis of monophyly of *Stenodactylus* + *Pseudoceramodactylus* (AU:0.461, SH:0.839, BF:0.647), monophyly of *Tropiocolotes* (AU:0.161, SH:0.495, BF:-0.530) or both concurrently (AU:0.153, SH:0.492, BF:1.589).

**Table 2:** **Statistical support for alternative hypotheses on *Stenodactylus *phylogeny**

		** ML framework**^**1**^		** Bayesian framework**^**2**^	
**Tree**	**-log likelihood**	**AU *P***	**SH *P***	** HME**	**log**_**10 **_**BF**
Unconstrained tree	15180.095955			−15175.4907	
Monophyly of *Stenodactylus+Pseudoceramodactylus*	15180.877129	0.461	0.839	−15175.0633	0.647
Monophyly of *Tropiocolotes*	15183.765511	0.161	0.495	−15175.6215	−0.530
Monophyly of *Stenodactylus+Pseudoceramodactylus* and *Tropiocolotes*	15183.696084	0.153	0.492	−15177.9132	1.589
Monophyly of African species	15192.711115	**0.029**	0.123	−15190.3457	**7.221**
Monophyly of *S. petrii*	15189.220967	**0.036**	0.210	−15181.4116	**2.578**

All topological tests are done versus the unconstrained (best) tree. Values in bold indicate statistically significant results.

^1^ML: Maximum likelihood; AU: Approximately Unbiased test (Shimodaira, 2002); SH: Shimodaira & Hasegawa (1999) test. *P* < 0.05 suggests that the two solutions are significantly different.

^2^HME: The harmonic mean of sampled likelihoods as estimated by Tracer. BF: Bayes Factor. A log_10_ Bayes factor > 2 indicates decisive evidence for statistically significant difference between solutions.

Within *Stenodactylus*, three well supported clades are revealed (see Figure 2): (i) clade A, formed by the Arabian species *S. pulcher* Anderson, 1896 [36], *S. arabicus* and the divergent lineage *S. cf. arabicus*, (ii) clade B, that includes five Arabian species (*S. leptocosymbotes* Leviton and Anderson, 1967 [83], *S. doriae*, *S. slevini* Haas, 1957 [37], *S. grandiceps* Haas, 1952 [84] and *S. affinis* (Murray, 1884) [85] grouped in 2 sub-clades, and (iii) clade C, formed by the four African species (*S. petrii, S. stenurus* Werner, 1899 [86], *S. mauritanicus* Guichenot, 1850 [87] and *S. sthenodactylus* (Lichtenstein, 1823) and the southwest Arabian endemic *S. yemenensis* Arnold, 1980 [31].

Clade A is sister to the remaining species of the genus and includes the two morphologically similar but highly divergent species *S. pulcher* and *S. arabicus* (*p*-distance 12S: 12.5% and 16S: 14.5%) (Additional file 3: Table S2a). Genetic variability within *S. arabicus* is very high and includes two reciprocally monophyletic deep lineages (*p*-distance 12S: 7.7% and 16S: 5.0%) (Additional file 3: Table S2c), one of them restricted to the Sharqiya Sands (formerly Wahiba Sands) in Oman, hereafter referred to as *S. cf. arabicus*, and the other one covering the rest of the distribution range of the species. Network analysis of the nuclear gene fragments *c-mos* and *RAG*-2 shows that for the former all alleles are unique for each lineage and all but one for the latter (Figure 3).

Clade B is well supported and groups *S. doriae* and *S. leptocosymbotes* in sub-clade B1, while *S. slevini*, *S. grandiceps* and *S. affinis* in B2. Phylogenetic relationships are not completely resolved in the latter. Genetic distances between these five species are among the lowest in the genus (Additional file 3: Table S2a). Nuclear network analyses (Figure 3) reveal only unique alleles in the *c-mos* gene fragment for all five species, while there is some allele sharing in *RAG*-2 between *S. doriae* and *S. leptocosymbotes*.

Finally, clade C consists of three sub-clades, two African and one Arabian. The North African sub-clade C1 braches first, and the Arabian *S. yemenensis* is sister to sub-clade C3 formed by the two North African species *S. mauritanicus* and *S. sthenodactylus,* making the group of North African *Stenodactylus* species paraphyletic. Topological constraint analyses indicate that the alternative hypothesis of monophyly of the North African species is rejected by the AU and BF tests (AU:0.029, SH:0.123, BF:7.221) (Table 2).

In sub-clade C1, *S. stenurus* is nested within *S. petrii*, rendering the latter paraphyletic. The results of the topological constraint analysis in which *S. petrii* was forced monophyletic show that this hypothesis is rejected by both AU and BF tests (AU:0.036, SH:0.210, BF:2.578) (Table 2). Network analysis shows that *S. stenurus* lacks unique alleles in both nuclear markers (Figure 3). The level of intraspecific genetic variability within *S. petrii* (Additional file 3: Table S2b) is very high: the uncorrected *p*-distances between specimens from Egypt and Israel, and the remaining *S. petrii* specimens sampled for this study is 7.2% and 6.0% for the 12S and 16S mitochondrial markers, respectively (Additional file 3: Table S2c). Nuclear networks indicate that all six c-*mos* and four out of six *RAG*-2 alleles investigated are unique to this former lineage of *S. petrii* (Figure 3).

In sub-clade C3, the two North African species *S. sthenodactylus* and *S. mauritanicus* are reciprocally monophyletic and highly divergent (*p*-distance 12S: 10.9% and 16S: 7.2%) (Additional file 3: Table S2a). The former is highly variable (*p*-distance: 12S 4.7% and 16S 3.2%) (Additional file 3: Table S2b) and presents three deep lineages that follow a clear geographical pattern (Figures 1 and 2), grouping animals from: 1.- northern Egypt, Israel and Jordan; 2.- south, southeast Egypt and Kenya; 3.- all the animals from Libya, Algeria, Tunisia, Western Sahara and Mauritania, although a single specimen from NE Egypt (loc. 127 in Figure 1, Siwa Oasis) is also part of this latter clade. The results of the network analyses also show a differentiation between these three lineages. In c-*mos* (Figure 3), in the first lineage 7 out of 10 alleles are unique, in the second lineage 2 out of 6 and in the third lineage 9 out of 16, while in *RAG*-2, 3 out of 10, 2 out of 8 and 10 out of 12 are unique, respectively (Figure 3). Genetic variability within *S. mauritanicus* is slightly higher than in *S. sthenodactylus* (*p*-distance: 12S 4.7% and 16S 4.3%) (Additional file 3: Table S2b) and six different mitochondrial lineages with geographic structure are found: 1.- easternmost part of Libya and Egypt; 2.- central Libya; 3.- Tunisia; 4.- Northern Morocco; 5.- two very divergent samples from southeastern Morocco; and 6.- all the southern Morocco plus Western Sahara samples (see Figure 1 and Additional file 2: Figure S1).

### Estimation of divergence times

Convergence was confirmed examining the likelihood and posterior trace plots of the two runs with Tracer v.1.5. Effective sample sizes of the parameters were above 200, indicating a good representation of independent samples in the posterior. The estimated divergence times are illustrated in Figure 2 and the chronogram can be seen in Supplementary Figure 2 (Additional file 4: Figure S2). Diversification within *Stenodactylus* initiated 29.5 Ma ago (95% HPD: 20.7-39.2). In clade A, the split between *S. pulcher* and *S. arabicus* is dated back to 17.3 Ma (95% HPD: 11.3-23.6). The separation between the ancestors of clades B and C dates back to 21.8 Ma (95% HPD: 15.4-29.1), while diversification within these two clades started 11.0 Ma (95% HPD: 7.4-15.0) and 19.3 Ma (95% HPD: 13.1-25.5) ago, respectively.

Posterior mean rates for the 12S and 16S mitochondrial gene fragments were estimated at 0.00701 and 0.00642 substitutions per lineage per million years, respectively (or divergence rate: 1.402% and 1.284%). The posterior rates for the nuclear fragments, c-*mos* and *RAG*-2, were 0.00052 and 0.00060 respectively, more than 10 times lower than the mitochondrial ones. The 12S mitochondrial rate concords extremely well with the average rate for the same mitochondrial gene for three Canary Island reptile groups (*Gallotia, Tarentola* and *Chalcides*; 0.00755 for the 12S gene) as estimated by Carranza and Arnold (2012) [44].

### Ancestral area reconstruction

**Figure 4:**
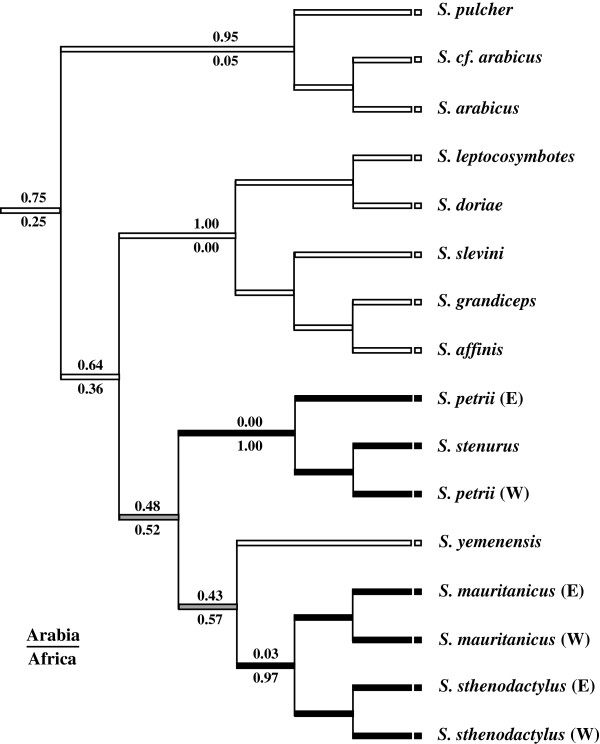
**Ancestral area reconstruction.** The tree figure illustrates the parsimony reconstruction, while numbers above and below nodes correspond to ML probabilities for character states. Black and white colors correspond to Africa and Arabia respectively, and grey color indicates equivocal nodes.

Reconstruction of the ancestral areas of *Stenodactylus* species was done in a parsimony framework based on the topology of the phylogeny presented in Figure 2. The analysis indicates that the reconstruction of the area for some of the ancestors is equivocal (see Figure 4). These are the common ancestor of clade C, formed by all North African species and *S. yemenensis*, and the ancestor of the latter and the sister species *S. sthenodactylus*/*S. mauritanicus*. Reconstructions using accelerated transformation (ACCTRAN) or delayed transformation (DELTRAN) optimizations support an identical number of events involving Arabia and Africa, but the direction of events is different. ML-based reconstruction, considering branch-length information, with the best-fit Markov k-state 1-parameter model also provided results with fairly similar probabilities for the two states in the aforementioned nodes (Figure 4).

## Discussion

This constitutes the first phylogenetic study using a complete sampling of *Stenodactylus* taxa and including 207 specimens from across the entire distribution range of North Africa and Arabia (Figure 1). This has enabled a robust phylogenetic reconstruction (see Figure 2 and Additional file 2: Figure S1), the uncovering of intraspecific diversity and, in some cases, the unveiling of interesting distribution patterns (see below). The phylogenetic results show a high level of support in most of the nodes and a striking agreement with the phylogenetic analyses of *Stenodactylus* by Arnold (1980) [31], based on morphological data, increasing our confidence that the recovered topology represents the true evolutionary history of the genus.

### Monophyly of *Stenodactylus*

Despite the general concordance between morphological and phylogenetic conclusions, one important discrepancy is observed: while morphology supports the inclusion of *P. khobarensis* in the genus *Stenodactylus*, the results of our molecular analyses indicate that *Pseudoceramodactylus* and *Stenodactylus* are not even sister genera (Figure 2). Kluge (1967) [39] transferred *P. khobarensis* to the genus *Stenodactylus* based on a “large number of external (meristic and mensural) and internal morphological similarities”, including relevant characters like the phalangeal reduction to a formula of 2.3.3.4.3 on both fore and hind limbs and a very high scleral ossicle number (20–28). Arnold (1980) [31], despite pointing out some unique scale characters of *P. khobarensis*, retained it in *Stenodactylus* and considered the scalation characters as “convincing pointers to holophyly”. However, according to a recent molecular analysis of the group by Fujita and Papenfuss (2011) [40] based on independent samples and sequences of different mitochondrial and nuclear regions, two representatives of *Tropiocolotes* branched between *P. khobarensis* and the six species of *Stenodactylus* included in the analysis (see Figure 1 of [40]). In order to deal with the non-monophyly of *Stenodactylus*, the genus *Pseudoceramodactylus* was resurrected. This pattern is repeated and further investigated in our study, with a complete taxon sampling of *Stenodactylus* and the inclusion of a greater number of representatives of *Tropiocolotes*, resulting in the splitting of the latter genus into two groups, a surprising but not strongly supported, albeit consistent, result.

We performed a series of constraint analyses in which *Stenodactylus* and *Pseudoceramodactylus* were forced to form a monophyletic group. Results clearly show that our dataset cannot reject the alternative hypothesis of a monophyletic *Stenodactylus + Pseudoceramodactylus* group (Table 2). In order to further investigate this, the dataset of Fujita and Papenfuss (2011) [40] was subjected to the same ML topological tests, but also could not reject the alternative hypothesis of monophyly of *Stenodactylus + Pseudoceramodactylus* (AU *P* = 0.074; SH *P* = 0.092). In view of the confusing molecular evidence and taking into account the morphological data, we think that the resurrection of *Pseudoceramodactylus* was precipitated, but in the meanwhile, this change accommodates for both the paraphyly reported by Fujita and Papenfuss and confirmed here, and the hypothesis of monophyly of *Stenodactylus + Pseudoceramodactylus*. We recommend not performing any further changes at the generic level before an in-depth revision clarifies the evolutionary relationships between the genera *Stenodactylus*, *Pseudoceramodactylus* and *Tropiocolotes*.

### Systematics and evolution

The well-supported clade A is formed by the morphologically similar *S. pulcher*, *S. arabicus* and the lineage *S. cf. arabicus* and, according to the inferred dates, the split between the former and the two latter species dates back to approximately 17 Ma ago (95% HPD: 11.3-23.6) (Figure 2). On the one hand, variability within *S. pulcher* is very low, probably as a result of the two specimens analyzed being from very close localities. On the other hand, the *S. cf. arabicus* lineage from the Sharqiya Sands (formerly Wahiba Sands), as already highlighted by Fujita and Papenfuss (2011) [40], is genetically very distinct from all other populations of *S. arabicus* included in our study in both mitochondrial and nuclear markers (Additional file 3: Table S2c and Figure 2), where almost all alleles are lineage-specific (see Results and Figure 3). This supports the idea that the Sharqiya Sands are isolated and surrounded by some areas of unsuitable habitat for sand dune specialists like this species [88-90]. Further morphological and molecular studies including more specimens from putative contact zones and faster nuclear markers are expected to give *S. cf. arabicus* formal recognition.

Clade B is well-supported (ML 100%, BI 1.0) and was also recovered by the morphological analysis of Arnold (1980) [34]. *Stenodactylus doriae* and *S. leptocosymbotes* are reciprocally monophyletic and form the relatively well-supported sub-clade B1 (Figure 2). Our molecular results agree with the results of the morphological analysis by Arnold (1980) [31], who also recovered the two species as sister taxa based on three synapomorphies. The two species diverged approximately 7.0 Ma ago (95% HPD: 4.2-10.1) (Figure 2) and, like the two North African sister species *S. sthenodactylus* and *S. mauritanicus*, they are ecologically distinct. *Stenodactylus leptocosymbotes* is an arid-adapted species that lives on relatively hard, although usually sandy, substrates being replaced by its sister species, *S. doriae*, on soft, wind-blown sand [34,91]. Thanks to its morphological and physiological adaptations, the latter is able to live in hyper-arid sand dune environments like for example the Eastern Rub al Khali [92], one of the largest and driest sand deserts in the world [93]. Given the clear morphological and ecological differences between these two species and the apparent absence of morphologically intermediate individuals [31,34], it seems reasonable to deduce that allele sharing in *RAG-*2 (see Results), which is limited to the ancestral allele, is the result of incomplete lineage sorting rather than ongoing gene flow between the two species. Variability within *S. leptocosymbotes* is rather low (Additional file 3: Table S2b) and the number of samples included permit to observe only moderate geographical structuring (Figures 1 and 2, Additional file 2: Figure S1). In contrast, *S. doriae*, shows a higher level of genetic differentiation, with the Sharqiya Sands lineage being quite divergent (Additional file 3: Table S2c and Figure 2), as already mentioned by Fujita and Papenfuss (2011) [40].

Sister to sub-clade B1 is a group composed by *S. slevini*, *S. grandiceps* and *S. affinis*, for which support is relatively low (ML 62, BI = 0.95). The topology within this sub-clade differs from the morphological hypothesis of Arnold (1980) [34], which supported the following relationship: (*S. grandiceps* (*S. affinis* (*S. slevini* (*S. leptocosymbotes*, *S. doriae*)))). *Stenodactylus slevini* is the only member of the group with two divergent lineages, one limited to Jordan and the other with representatives from East Arabia. Although the divergence based on mitochondrial data is clear (Additional file 2: Figure S1), there is no supporting nuclear data available (Figure 3), and no obvious morphological differences (pers. obs.). With the only exception of the soft wind-blown sand specialist *S. doriae*, all remaining representatives of clade B plus two other species, the African *S. sthenodactylus* and the Arabian *S. yemenensis*, appear to occupy rather similar spatial niches. These six species are adapted to living on relatively hard ground, coarse sandy planes, large wadis and sandy substrates and, based on their head dimensions, probably feed on similar-sized prey [31,32,34]. As a consequence of that, these species rarely coexist and have largely allopatric distribution ranges, while in places where they coincide they are not syntopic [31,33,34]. The analysis of the nuclear allele networks (Figure 3) indicate that the morphologically and ecologically similar and phylogenetically closely related *S. leptocosymbotes*, *S. slevini*, *S. grandiceps* and *S. affinis* do not share a single allele in the c-*mos* and *RAG*-2 genes analyzed, even though the results of the calibration analyses suggest that *S. grandiceps* and *S. affinis* diverged later (6.7 Ma ago; 95% HPD: 4.1-9.3) than other lineages for which extensive allele sharing in the *RAG*-2 has been identified (*S. doriae* and *S. leptocosymbotes*; see above and Results). These differences of the level of lineage sorting in some of the morphologically well-recognized species may also be the result of differences in effective population sizes, which affect the lineage coalescence time [94].

In sub-clade C1, *S. petrii* is grouped together with the North African endemic *S. stenurus* that branches inside it (Figure 2). As a result, *S. petrii* is paraphyletic and constitutes the only exception among the otherwise monophyletic *Stenodactylus* species. The results of the topological tests (Table 2) indicate that our dataset most probably rejects the monophyly of this species (AU:0.036, SH:0.210, BF:2.578). *Stenodactylus stenurus* was described by Werner (1899) [86] and synonymized ten years later by the same author [95]. It remained in synonymy until Kratochvil et al. (2001) [96] recognized it as a valid species, based on a multivariate analysis of several metric and scalation characters. It is noteworthy that the representative of *S. stenurus* included in our analysis is one of the specimens used by Kratochvil et al. (2001) [96] in their study.

The highly divergent lineage that includes specimens from Egypt and Israel (see Results) is estimated to have split from specimens further west in Algeria, Morocco, Western Sahara and Mauritania approximately 6.1 Ma ago (95% HPD: 3.9-8.6) (Figure 2). In fact, the northern Sinai populations of *S. petrii* have been reported to be morphologically distinct and, as a result of that, were considered a different species (*S. elimensis*) by Barbour (1914) [97], now under the synonymy of *S. petrii*[31,98]. Yet, specimens from this area included in our analyses do not present considerable genetic differences with the rest of the Egyptian and Israeli specimens (Figures 1 and 2, Additional file 2: Figure S1). It should be pointed out that the type locality of *S. petrii* is Egypt and, thus, this lineage represents the 'true' *S. petrii*. The pattern in the nuclear genes, with numerous unique alleles for this lineage (Figure 3), contrasts with the situation in *S. stenurus* that lacks unique alleles. This suggests that further analyses and a thorough taxonomic revision including more samples of *S. petrii*, especially from not sampled areas of Algeria and Libya, and mainly *S. stenurus* will be necessary in order to evaluate the status of the populations assigned to the two species. With this evidence it will be possible to differentiate between a single species with high genetic variability (*petrii*), two species (*petrii* in the East and *stenurus* in the West) or three species, if *stenurus* proves to be distinct from the more western forms.

The two North African species of sub-clade C3, *S. sthenodactylus* and *S. mauritanicus*, are shown to be reciprocally monophyletic and highly divergent (Additional file 3: Table S2a), while their separation dates back to approximately 10.0 Ma (95% HPD: 6.6-13.7) (Figure 2). These results help to clarify the status of these two taxonomically controversial taxa that were treated as two different subspecies by Loveridge (1947) [99] and Sindaco and Jeremcenko (2008) [33], as the same monotypic species by Arnold (1980) [31] and that were finally considered as full species by Baha el Din (2006) [98], who found them in sympatry at particular localities in northern Egypt. As observed by Baha el Din (2006) [98], although these two sister species can be morphologically similar and share similar habits, they are ecologically different. *Stenodactylus mauritanicus* is restricted to fairly mesic coastal semi-desert under the influence of the Mediterranean (see Figure 1), where it inhabits flat rock-strewn sand and gravel plains with fairly good vegetation cover. On the contrary, *S. sthenodactylus* inhabits areas of the Sahara that are far more arid and inhospitable than the ones of its sister species (see Figure 1), being the only vertebrate to be readily found in some parts of the Western Desert of Egypt [98]. It prefers gravelly and coarse sandy plains and large wadis and, although the species is typical of hard coarse substrates, it sometimes penetrates some dune areas [98].

The distributions of these two species, as introduced by the present study, give insights into the controversial taxonomic status and frequent misidentification of the two forms [99]. Our analysis concludes that *S. sthenodactylus* extends west from the Middle East and Egypt, previously thought to be its eastern limit, across the Sahara and into Mauritania (Figure 1). *Stenodactylus mauritanicus* is confirmed to be present in Egypt [98] and has a wide, almost continuous distribution roughly along the northern margin of the Sahara desert. The two species are found in sympatry or in close proximity in Egypt and coastal Mauritania, yet retain distinct mtDNA lineages and exhibit only limited allele sharing in the nuclear markers, most of which is due to sharing of ancestral alleles and hence is likely to represent incomplete lineage sorting (see Figure 3).

*Stenodactylus sthenodactylus* presents high variability, both at genetic (see Results) and morphological levels [31]. Its three deep lineages are estimated to have diverged approximately 4.8 Ma ago (95% HPD: 2.8-6.9) (Figure 2). According to Baha el Din (2006) [98], some morphological characters appear to correlate with environmental factors, with populations from hyper-arid places showing a very slender body, less contrasting pattern and tubular nostrils, while populations from more mesic areas being usually more robust, with thick limbs, big heads and marked pattern [31,36,98]. The populations from coastal regions in southeast Egypt are especially distinct and, according to Baha el Din (2006) [98], they resemble specimens of *S. s. zavattarii* from Kenya, which Loveridge (1957) [100] synonymized with *S. sthenodactylus*. Two specimens of this form were included in our phylogenetic analyses (see Figure 2 and Additional file 2: Figure S1), and indeed they belong to a clade with samples from south and southeast Egypt. These results suggest that some of the morphological variability between populations of *S. sthenodactylus* may also be supported by molecular data. A nomenclatural revision of North African *Stenodactylus* (work in progress) is essential for stability before any changes are performed, while further work focused on the contact zones between the three lineages and combining detailed morphological analyses with additional nuclear data is needed in order to determine if they deserve formal recognition.

On the other hand, the high genetic variability within *S. mauritanicus* (Figure 2 and Additional file 3: Table S2b) does not seem to correlate with differences in morphology. This species is fairly uniform morphologically, with populations from the West being a bit larger than Egyptian ones but generally maintaining the same proportions, pattern and scalation across most of its distribution range [98]. Nevertheless, the intra-specific divergence is estimated to date back to 6.6 Ma ago (95% HPD: 4.0-9.5) and the six mitochondrial lineages present a clear geographical pattern (Figure 1 and Additional file 2: Figure S1). The relationship between these lineages, however, is not clear and neither is any structure observed in the nuclear alleles (Figure 3), both facts being mirrored in the low-supported nodes of the concatenated phylogeny (Figure 2).

### Origin, biogeography and diversification of *Stenodactylus*

Reconstruction of ancestral areas with both parsimony and ML methods (Figure 4) suggests that the genus *Stenodactylus* originated in Arabia approximately 30 Ma ago (95% HPD: 20.7-39.2) (Figure 2), a time of high geological instability as a result of the onset of major seismic and volcanic events in the general area of Ethiopia, northeast Sudan and southwest Yemen [101]. These major volcanic and tectonic events, centered over the Afar region, marked the onset of the formation of some of the most relevant and complex physiographical features in the contact zone between Africa and Arabia, like the Gulf of Aden, the Red Sea and the elevation of the Afro-Arabian rift-flanks to heights above 3600 m [1,101,102].

The tempo and mode of the deep splits in *Stenodactylus* bear a striking resemblance to the basal splits that occurred in the African-Eurasian snake genus *Echis*[13,103], which suggests a common biogeographical pattern for both groups. The distribution of the members of Arabian clade B (*S. doriae*, *S. leptocosymbotes*, *S. slevini*, *S. grandiceps*, *S. affinis*) and the mainly African clade C (*S. petrii*, *S. stenurus*, *S. yemenensis*, *S. mauritanicus*, *S. sthenodactylus*) (Figures 1 and 2) extend primarily on the opposite sides of the Red Sea, mimicking the situation of the sister taxa *E. coloratus* (mainly Arabian) and *E. pyramidum* (mainly African). The split between these two *Stenodactylus* groups dates back to 21.8 Ma ago (95% HPD: 15.4-29.1) (Figure 2), which roughly coincides with the split between *E. coloratus* and *E. pyramidum* calculated at approximately 19.4 Ma ago. The dates of these phylogenetic events follow a well-studied phase of volcanism and strong rifting initiated at approximately 24 Ma ago, that appeared in an almost synchronous way throughout the entire Red Sea [1]. Therefore, it is possible that the formation of the Red Sea acted as a vicariant event separating the aforementioned clades of *Stenodactylus*, as also suggested by Pook et al. (2009) [13] for the genus *Echis*. The agamid lizards of the genus *Uromastyx*[25] is yet another group that could have been affected by such an event, although in this case the split between the Arabian and African clades seems to have happened later, at 11–15 Ma ago. Amer and Kumazawa (2005) [25] attributed this split to a dispersal event from Arabia into North Africa, coinciding with climatic changes towards aridity in this latter area, rather than to vicariance. However, since earlier dates had also been calculated for the split between African and Arabian *Uromastyx* that coincide with the inferred dates for *Stenodactylus* and *Echis* (18 Ma ago; [104]), a reassessment of the calibration dates of *Uromastyx* using relaxed clock methods like the ones applied by Pook et al. (2009) [13] and in the present study seems necessary (work in progress).

The split between the Arabian *S. yemenensis* and the ancestor of the African *S. mauritanicus* and *S. sthenodactylus* on either sides of the Red Sea also parallels the splits between Arabian and African sister clades of the *E. pyramidum* complex [13] and *Uromastyx ocellata* and *U. ornata*[25]. Although the divergence time estimate for the *Stenodactylus* members (15.4 Ma ago (95% HPD: 10.5-20.8), Figure 2) predates the ones of the other two groups by almost 7 Ma, the split between African and Arabian lineages might be explained by the complex geology of the Red Sea. Several recurrent episodes during the Miocene caused the desiccation and refilling of this tectonically active rifting area [1,105] and provoked the severing of the land bridges that had existed after the initial formation of the Red Sea in the early Miocene. So, the separation between *S. yemenensis* and the ancestor of *S. mauritanicus* and *S. sthenodactylus* was probably also the result of vicariance, similarly to *Echis* and *Uromastyx*. After this event, *S. yemenensis* would have remained isolated at the coastal side of the southern Arabian highlands (Figures 1 and 2).

In Arabia, an example of a similar biogeographical pattern caused by a different biogeographical process is the case of the ecologically similar sister species of clade A, *S. pulcher* and *S. arabicus* (including *S. cf. arabicus*), which, according to the results (Figure 2) and the geological data available, are hypothesized to result from vicariance caused by the uplift of the Yemen Mountains approximately 18 Ma ago [1,101,102]. The splits within clade B, however, seem more difficult to interpret, as little information is available on the geological and climatic history of the interior of Arabia. A general pattern could be proposed with a first North–South split between the ancestors of *S. doriae*, *S. leptocosymbotes* and *S. slevini*, *S. grandiceps*, *S. affinis*, respectively, followed by the posterior range expansion of some of these species. Interestingly, in Arabia, even though evidence exists for an increase in aridification [106], it has been hypothesized that at the same time an important river system, as evidenced by the fluvial sediments, could characterize the interior of the peninsula [93,107]. Such dynamic scenery could be responsible for the rapid diversification within clade B, having caused fragmentation of the distribution range of the ancestor(s) and the different lineages to split allopatrically.

The onset of diversification in clade B coincides in time with the split between the African *S. mauritanicus* and *S. sthenodactylus* in sub-clade C3 (Figure 2). These speciation events match very closely the estimates of the formation, in the late Miocene, of a major east-Antarctic ice sheet with its associated polar cooling, which triggered the aridification of mid-latitude continental regions and a shift in North Africa from forest to dry open woodlands and savannahs [4,20,108]. The two North African forms, *S. mauritanicus* and *S. sthenodactylus*, seem to have diverged in ecological niche, with one form adapted to mesic environments and the other occupying much dryer areas, respectively. It has been proposed that the gradual increase in aridity that took place in northern Africa during the late Miocene accelerated the diversification process in reptile faunas [21]. The estimated divergence times of the North African *Stenodactylus* seem to corroborate a common emerging pattern among European biota, according to which the speciation events in many reptile and amphibian groups do not coincide with the accentuated environmental instability during the Pleistocene, but rather date back into the Miocene and proceeding through the Quaternary, when many species and populations originated [109,110].

It has been suggested that 18 Ma ago, Africa connected with Eurasia through the closure of the Eastern Mediterranean seaway (the *Gomphotherium* land bridge) [15]. This land bridge later became disconnected temporarily but it has been continuously present since approximately 15 Ma ago. It is interesting to notice that, despite the existence of a continuous passage between Arabia and Eurasia, our phylogeny suggests that colonization of Eurasia by members of the genus *Stenodactylus* occurred much later and was very restricted geographically. In fact, only two *Stenodactylus* species extend their ranges into Eurasia (*S. affinis* and *S. doriae*). From these two, only samples of *S. affinis* from Eurasia (Iran) were available, while for the other species a specimen from neighboring Kuwait was included. In both species, however, the low intraspecific genetic variability suggests that the colonization of Eurasia was a very recent event (Figure 2 and Additional file 3: Table S2b). One possible explanation of this biogeographical pattern may be the existence of ecologically and morphologically very similar forms in Iran like *Crossobamon* (formerly a member of *Stenodactylus*[39]) and *Agamura*, which may compete with *Stenodactylus* and therefore may have not allowed it to expand further outside the narrow coastal strip in southwestern Iran (Arnold, 1980). This situation is completely different than the one in North Africa, where no ecological analogs to *Stenodactylus* seem to exist and therefore several of its species are found across an area of more than 10 million Km^2^[31,33,98,111,112].

## Conclusions

The analyses presented in this study, based on a multi-locus dataset that derives from a complete sampling of the 12 species of the genus *Stenodactylus*, reveal the existence of three clades with deep divergences within *Stenodactylus* and high intraspecific variability in some species, while the estimation of divergence times allows for biogeographical interpretations. The geckos *Stenodactylus* originated in Arabia 30 Ma ago. In clade A, the split between the two species is hypothesized to have resulted from vicariance caused by the uplift of the Yemen Mountains approximately 18 Ma ago. *Stenodactylus cf. arabicus* from the Sharqiya Sands constitutes a genetically and morphologically distinct lineage. In clade B, rapid diversification seems to relate to climatic and geological instability in the late Miocene, but this hinders the reconstruction of robust phylogenetic relationships between some species. The Sharqiya Sands host yet another divergent lineage, that of the species *S. doriae*. In clade C, the split between *S. yemenensis* and sub-clade C3 is hypothesized to relate to the recurrent episodes of the desiccation and refilling of the Red Sea, during the Miocene. An interesting distribution pattern is revealed for the sister species *S. sthenodactylus* and *S. mauritanicus*, differing greatly from what was previously thought. Several speciation events in *Stenodactylus* are estimated to date back to the late Miocene, indicating that this was an important period for reptile diversification in this area. The split between clades B and C is attributed to the opening of the Red Sea in the Upper Miocene, acting as a vicariant agent. On the other hand, the formation of the connection between Africa and Eurasia seems to have had little effect on *Stenodactylus*, probably because of the existence of ecological analogs. On a taxonomic level, further studies are expected to resolve the systematics of the *S. petrii* - *S. stenurus* complex. Validity of the specific status of *S. mauritanicus* is confirmed with mitochondrial and nuclear data. Overall, this work unveils the evolutionary history of *Stenodactylus* geckos and highlights their use as a model in the study of the faunal interchanges between North Africa and Arabia and the evolutionary processes in these arid areas.

## Abbreviations

rRNA: ribosomal ribonucleic acid;c-mos: oocyte maturation factor Mos;RAG-2: Recombination activating gene 2;PCR: Polymerase chain reaction;ML: Maximum likelihood;BI: Bayesian Inference;AU: Approximately unbiased;SH: Shimodaira-Hasegawa;BF: Bayes factor;Ma: Megaannum;HPD: Highest posterior density

## Competing interests

The authors declare that they have no competing interests.

## Authors’ contributions

SC and ENA conceived the study. SC coordinated the study. All authors collected samples in the field and/or provided tissue samples. SC and MM assembled the data. MM obtained the sequences, carried out the analyses and drafted the manuscript. PAC contributed to improving the manuscript. MM and SC wrote the final manuscript. All authors read and approved the final manuscript.

## Supplementary Material

Additional file 1: Table S1 Information on the specimens used in the phylogenetic analyses. Click here for file

Additional file 2: Figure S1 BI tree of the genus *Stenodactylus* inferred using 12S and 16S mtDNA gene fragments. Description of data: Posterior probability values above 0.95 in the Bayesian Inference analysis are indicated next to the nodes with an asterisk, while numbers correspond to bootstrap support of the Maximum Likelihood analysis (only values above 70 are shown). The tree was rooted using *Hemidactylus frenatus*. Numbers in square brackets next to specimen code refer to Figure 1. Information on the samples included is shown in Additional file 1: Table S1. Click here for file

Additional file 3: Table S2 Uncorrected *p*-distances (pairwise deletion). Click here for file

Additional file 4: Figure S2 Chronogram obtained with BEAST inferred using all markers and 3 calibration points. Description of data: Chronogram obtained with relaxed uncorrelated lognormal clock and Yule model of speciation. Filled numbered circles correspond to calibration points described in Materials and Methods. The *x* axis is in million years and the bars indicate 95% HPD intervals. Information on the samples included is shown in Additional file 1: Figure S1. Click here for file

## References

[B1] Bosworth W Huchon P McClay K The Red Sea and Gulf of Aden Basins. J Afr Earth Sci 2005 43 334 378 10.1016/j.jafrearsci.2005.07.020

[B2] Carranza S Arnold EN Geniez P Roca J Mateo J Radiation, multiple dispersal and parallelism in the skinks, *Chalcides *and *Sphenops *(Squamata: Scincidae), with comments on *Scincus *and *Scincopus *and the age of the Sahara desert. Mol Phylogenet Evol 2008 46 1071 1094 10.1016/j.ympev.2007.11.018 18276164

[B3] Dean WRJ Nomadic Desert Birds 2004 Berlin, Heidelberg, New York: Springer Verlag

[B4] Flower BP Kennett JP The middle Miocene climatic transition: East Antarctic ice sheet development, deep ocean circulation and global carbon cycling Palaeogeogr Palaeoclimatol Palaeoecol 1994 108 537 555 10.1016/0031-0182(94)90251-8

[B5] Griffin DL Aridity and humidity: two aspects of the late Miocene climate of North Africa and the Mediterranean Palaeogeogr Palaeoclimatol Palaeoecol 2002 182 65 91 10.1016/S0031-0182(01)00453-9

[B6] Guiraud R Bosworth W Thierry J Delplanque A Phanerozoic geological evolution of Northern and Central Africa: an overview J Afr Earth Sci 2005 43 83 143 10.1016/j.jafrearsci.2005.07.017

[B7] Lourenço W Duhem B Saharo-Sindian buthid scorpions; description of two new genera and species from Occidental Sahara and Afghanistan ZooKeys 2009 14 37 54

[B8] Quezel P Analysis of the flora of Mediterranean and Saharan Africa Ann Mo Bot Gard 1978 65 479 534 10.2307/2398860

[B9] Yom-Tov Y Character displacement in the Psammophile Gerbillidae of Israel Oikos 1991 60 173 179 10.2307/3544863

[B10] Haq BU Hardenbol J Vail PR Chronology of fluctuating sea levels since the Triassic Science 1987 235 1156 1167 10.1126/science.235.4793.1156 17818978

[B11] Fernandes CA Rohling EJ Siddall M Absence of post-Miocene Red Sea land bridges: biogeographic implications J Biogeogr 2006 33 961 966 10.1111/j.1365-2699.2006.01478.x

[B12] Harzhauser M Kroh A Mandic O Piller WE Göhlich U Reuter M Berning B Biogeographic responses to geodynamics: a key study all around the Oligo-Miocene Tethyan Seaway Zoologischer Anzeiger-A Journal of Comparative Zoology 2007 246 241 256 10.1016/j.jcz.2007.05.001

[B13] Pook CE Joger U Stümpel N Wüster W When continents collide: phylogeny, historical biogeography and systematics of the medically important viper genus *Echis* (Squamata: Serpentes: Viperidae) Mol Phylogenet Evol 2009 53 792 807 10.1016/j.ympev.2009.08.002 19666129

[B14] Zhou L Su YCF Thomas DC Saunders RMK 'Out-of-Africa' dispersal of tropical floras during the Miocene climatic optimum: evidence from *Uvaria* (Annonaceae) J Biogeogr 2012 39 322 335 10.1111/j.1365-2699.2011.02598.x

[B15] Rögl F Paleogeographic Considerations For Mediterranean And Paratethys Seaways (Oligocene And Miocene) 1998 Wien: Annalen des Naturhistorischen Museums in 99A: 279-331

[B16] Kroepelin S Revisiting the age of the Sahara desert Science 2006 312 1138 1139 16728618 10.1126/science.312.5777.1138b

[B17] Schuster M Revisiting the age of the Sahara Desert Science 2006 312 1138 1139 10.1126/science.312.5777.1138b 16728618

[B18] Schuster M Duringer P Ghienne J-F Vignaud P Mackaye HT Likius A Brunet M The age of the Sahara desert Science 2006 311 821 10.1126/science.1120161 16469920

[B19] Swezey CS Revisiting the age of the Sahara desert Science 2006 312 1138 1139 16739253

[B20] Douady CJ Catzeflis F Raman J Springer MS Stanhope MJ The Sahara as a vicariant agent, and the role of Miocene climatic events, in the diversification of the mammalian order Macroscelidea (elephant shrews) Proc Natl Acad Sci 2003 100 8325 8330 10.1073/pnas.0832467100 12821774 PMC166228

[B21] Fu J Toward the phylogeny of the family Lacertidae–Why 4708 base pairs of mtDNA sequences cannot draw the picture Biol J Linn Soc 2000 71 203 217

[B22] Guillaumet A Crochet PA Pons JM Climate-driven diversification in two widespread *Galerida *larks BMC Evol Biol 2008 8 32 10.1186/1471-2148-8-32 18230151 PMC2275783

[B23] Camargo A Sinervo B Sites JW Jr Lizards as model organisms for linking phylogeographic and speciation studies Mol Ecol 2010 19 3250 3270 10.1111/j.1365-294X.2010.04722.x 20618905

[B24] Kapli P Lymberakis P Poulakakis N Mantziou G Parmakelis A Mylonas M Molecular phylogeny of three *Mesalina *(Reptilia: Lacertidae) species (*M. guttulata, M. brevirostris *and *M. bahaeldini*) from North Africa and the Middle East: another case of paraphyly? Mol Phylogenet Evol 2008 49 102 110 10.1016/j.ympev.2008.06.016 18644456

[B25] Amer SAM Kumazawa Y Mitochondrial DNA sequences of the Afro-Arabian spiny-tailed lizards (genus *Uromastyx*; family Agamidae): phylogenetic analyses and evolution of gene arrangements Biol J Linn Soc 2005 85 247 260 10.1111/j.1095-8312.2005.00485.x

[B26] Carranza S Arnold EN Mateo JA Geniez P Relationships and evolution of the North African geckos, *Geckonia* and *Tarentola* (Reptilia: Gekkonidae), based on mitochondrial and nuclear DNA sequences Mol Phylogenet Evol 2002 23 244 256 10.1016/S1055-7903(02)00024-6 12069554

[B27] Carranza S Arnold EN Pleguezuelos JM Phylogeny, biogeography, and evolution of two Mediterranean snakes, *Malpolon monspessulanus* and *Hemorrhois hippocrepis* (Squamata, Colubridae), using mtDNA sequences Mol Phylogenet Evol 2006 40 532 546 10.1016/j.ympev.2006.03.028 16679033

[B28] Fonseca MM Brito JC Rebelo H Kalboussi M Larbes S Carretero MA Harris DJ Genetic variation among spiny-footed lizards in the *Acanthodactylus pardalis* group from North Africa African Zoology 2008 43 8 15 10.3377/1562-7020(2008)43[8:GVASLI]2.0.CO;2

[B29] Gonçalves DV Brito JC Crochet PA Geniez P Padial JM Harris DJ Phylogeny of North African *Agama* lizards (Reptilia: Agamidae) and the role of the Sahara desert in vertebrate speciation Mol Phylogenet Evol 2012 64 582 591 10.1016/j.ympev.2012.05.007 22634241

[B30] Fitzinger LJ Neue Classification Der Reptilien Nach Ihren Natürlichen Verwandtschaften: Nebst Einer Verwandtschafts-Tafel Und Einem Verzeichnisse Der Reptilien-Sammlung Des KK Zoologischen Museum's Zu Wien 1826 Wien: JG Heubner

[B31] Arnold EN Reptiles of Saudi Arabia: a review of the lizard genus *Stenodactylus* (Reptilia: Gekkonidae) Fauna of Saudia Arabia 1980 2 368 404

[B32] Arnold EN Little-known geckoes (Reptilia: Gekkonidae) from Arabia with descriptions of two new species from the Sultanate of Oman The Scientific Results of the Oman Flora and Fauna Survey 1975 1977 81 110

[B33] Sindaco R Jeremcenko VK The Reptiles Of The Western Palearctic 2008 Latina (Italy): Edizioni Belvedere

[B34] Arnold EN Ecology of lowland lizards in the eastern United Arab Emirates J Zool 1984 204 329 354

[B35] Blanford WT Descriptions of new reptilia and amphibia from Persia and Baluchistan The Annals and Magazine of Natural History, London 1874 4 31 35

[B36] Anderson J A Contribution To The Herpetology Of Arabia: With A Preliminary List Of The Reptiles And Batrachians Of Egypt 1896 London: RH Porter

[B37] Haas G Some amphibians and reptiles from Arabia Proc Calif Acad Sci 1957 29 47 86

[B38] Bauer AM Russell AP Pedal specialisations in dune-dwelling geckos J Arid Environ 1991 20 43 62

[B39] Kluge AG Higher taxonomic categories of gekkonid lizards and their evolution Bull Am Mus Nat Hist 1967 135 1 60

[B40] Fujita MK Papenfuss TJ Molecular systematics of *Stenodactylus* (Gekkonidae), an Afro-Arabian gecko species complex Mol Phylogenet Evol 2011 58 71 75 10.1016/j.ympev.2010.10.014 21035555

[B41] Arnold EN Relationships, evolution and biogeography of Semaphore geckos, *Pristurus* (Squamata, Sphaerodactylidae) based on morphology Zootaxa 2009 2060 1 21

[B42] Arnold EN Vasconcelos R Harris DJ Mateo JA Carranza S Systematics, biogeography and evolution of the endemic *Hemidactylus* geckos (Reptilia, Squamata, Gekkonidae) of the Cape Verde Islands: based on morphology and mitochondrial and nuclear DNA sequences Zoologica Scripta 2008 37 619 636 10.1111/j.1463-6409.2008.00351.x

[B43] Carranza S Arnold EN Systematics, biogeography, and evolution of *Hemidactylus* geckos (Reptilia: Gekkonidae) elucidated using mitochondrial DNA sequences Mol Phylogenet Evol 2006 38 531 545 10.1016/j.ympev.2005.07.012 16154768

[B44] Carranza S Arnold EN A review of the geckos of the genus *Hemidactylus* (Squamata: Gekkonidae) from Oman based on morphology, mitochondrial and nuclear data, with descriptions of eight new species Zootaxa 2012 3378 1 95

[B45] Carranza S Arnold EN Mateo JA López-Jurado LF Long-distance colonization and radiation in gekkonid lizards, *Tarentola* (Reptilia: Gekkonidae), revealed by mitochondrial DNA sequences Proc R Soc London, Ser B 2000 267 637 10.1098/rspb.2000.1050 PMC1690580 10821607

[B46] Gamble T Bauer AM Colli GR Greenbaum E Jackman TR Vitt LJ Simons AM Coming to America: multiple origins of New World geckos J Evol Biol 2011 24 231 244 10.1111/j.1420-9101.2010.02184.x 21126276 PMC3075428

[B47] Feng J Han D Bauer AM Zhou K Interrelationships among Gekkonid Geckos inferred from mitochondrial and nuclear gene sequences Zoolog Sci 2007 24 656 665 10.2108/zsj.24.656 17824773

[B48] Gamble T Bauer AM Greenbaum E Jackman TR Out of the blue: a novel, trans-Atlantic clade of geckos (Gekkota, Squamata) Zoologica Scripta 2008 37 355 366 10.1111/j.1463-6409.2008.00330.x

[B49] Han D Zhou K Bauer AM Phylogenetic relationships among gekkotan lizards inferred from C-*mos* nuclear DNA sequences and a new classification of the Gekkota Biol J Linn Soc 2004 83 353 368 10.1111/j.1095-8312.2004.00393.x

[B50] Drummond AJ Ashton B Buxton S Cheung M Cooper A Heled J Kearse M Moir R Stones-Havas S Sturrock S Geneious v5. 1 2010 Available from http://www.geneious.com

[B51] Katoh K Toh H Recent developments in the MAFFT multiple sequence alignment program Brief Bioinform 2008 9 286 298 10.1093/bib/bbn013 18372315

[B52] Tamura K Peterson D Peterson N Stecher G Nei M Kumar S MEGA5: molecular evolutionary genetics analysis using maximum likelihood, evolutionary distance, and maximum parsimony methods Mol Biol Evol 2011 28 2731 2739 10.1093/molbev/msr121 21546353 PMC3203626

[B53] Akaike H Petrov BN, Csaki F Information theory and an extension of the maximum likelihood principle Second International Symposium on Information Theory 1973 Budapest (Hungary): Akademiai Kiado 267 281

[B54] Posada D jModelTest: phylogenetic model averaging Mol Biol Evol 2008 25 1253 10.1093/molbev/msn083 18397919

[B55] Huelsenbeck JP Ronquist F MRBAYES: Bayesian inference of phylogenetic trees Bioinformatics 2001 17 754 755 10.1093/bioinformatics/17.8.754 11524383

[B56] Ronquist F Huelsenbeck JP MrBayes 3: Bayesian phylogenetic inference under mixed models Bioinformatics 2003 19 1572 1574 10.1093/bioinformatics/btg180 12912839

[B57] Stamatakis A RAxML-VI-HPC: maximum likelihood-based phylogenetic analyses with thousands of taxa and mixed models Bioinformatics 2006 22 2688 10.1093/bioinformatics/btl446 16928733

[B58] Felsenstein J Confidence limits on phylogenies: an approach using the bootstrap Evolution 1985 39 783 791 10.2307/2408678 28561359

[B59] Stephens M Scheet P Accounting for decay of linkage disequilibrium in haplotype inference and missing-data imputation Am J Hum Genet 2005 76 449 462 10.1086/428594 15700229 PMC1196397

[B60] Stephens M Smith NJ Donnelly P A new statistical method for haplotype reconstruction from population data Am J Hum Genet 2001 68 978 989 10.1086/319501 11254454 PMC1275651

[B61] Flot JF Seqphase: a web tool for interconverting phase input/output files and fasta sequence alignments Mol Ecol Resour 2010 10 162 166 10.1111/j.1755-0998.2009.02732.x 21565002

[B62] Clement M Posada D Crandall KA TCS: a computer program to estimate gene genealogies Mol Ecol 2000 9 1657 1659 10.1046/j.1365-294x.2000.01020.x 11050560

[B63] Shimodaira H An approximately unbiased test of phylogenetic tree selection Syst Biol 2002 51 492 10.1080/10635150290069913 12079646

[B64] Shimodaira H Hasegawa M Multiple comparisons of log-likelihoods with applications to phylogenetic inference Mol Biol Evol 1999 16 1114 1116 10.1093/oxfordjournals.molbev.a026201

[B65] Shimodaira H Hasegawa M CONSEL: for assessing the confidence of phylogenetic tree selection Bioinformatics 2001 17 1246 10.1093/bioinformatics/17.12.1246 11751242

[B66] Suchard MA Weiss RE Sinsheimer JS Models for estimating bayes factors with applications to phylogeny and tests of monophyly Biometrics 2005 61 665 673 10.1111/j.1541-0420.2005.00352.x 16135017

[B67] Drummond AJ Rambaut A BEAST: Bayesian evolutionary analysis by sampling trees BMC Evol Biol 2007 7 214 10.1186/1471-2148-7-214 17996036 PMC2247476

[B68] Rambaut A Drummond AJ Tracer v1. 4 2007 [http://beast.bio.ed.ac.uk/Tracer]

[B69] Ho SYW Phillips MJ Cooper A Drummond AJ Time dependency of molecular rate estimates and systematic overestimation of recent divergence times Mol Biol Evol 2005 22 1561 1568 10.1093/molbev/msi145 15814826

[B70] Gamble T Bauer AM Greenbaum E Jackman TR Evidence for Gondwanan vicariance in an ancient clade of gecko lizards J Biogeogr 2008 35 88 104

[B71] Vasconcelos R Carranza S Harris DJ Insight into an island radiation: the *Tarentola* geckos of the Cape Verde archipelago J Biogeogr 2010 37 1047 1060 10.1111/j.1365-2699.2009.02254.x

[B72] Agustí J Cabrera L Garcés M Krijgsman W Oms O Parés JM A calibrated mammal scale for the Neogene of Western Europe State of the art. Earth-Science Reviews 2001 52 247 260 10.1016/S0012-8252(00)00025-8

[B73] Müller J A new fossil species of *Euleptes* from the early Miocene of Montaigu, France (Reptilia, Gekkonidae) Amphibia-Reptilia 2001 22 341 348 10.1163/156853801317050133

[B74] Abdrakhmatov KY Aldazhanov SA Hager BH Hamburger MW Herring TA Kalabaev KB Makarov VI Molnar P Panasyuk SV Prilepin MT Relatively recent construction of the Tien Shan inferred from GPS measurements of present-day crustal deformation rates Nature 1996 384 450 453 10.1038/384450a0

[B75] Macey JR Wang Y Ananjeva NB Larson A Papenfuss TJ Vicariant patterns of fragmentation among Gekkonid lizards of the Genus *Teratoscincus* produced by the Indian collision: a molecular phylogenetic perspective and an area cladogram for Central Asia Mol Phylogenet Evol 1999 12 320 332 10.1006/mpev.1999.0641 10413626

[B76] Tapponnier P Mattauer M Proust F Cassaigneau C Mesozoic ophiolites, sutures, and large-scale tectonic movements in Afghanistan Earth Planet Sci Lett 1981 52 355 371 10.1016/0012-821X(81)90189-8

[B77] Guillou H Carracedo JC Torrado FP Badiola ER K-Ar ages and magnetic stratigraphy of a hotspot-induced, fast grown oceanic island: El Hierro, Canary Islands J Volcanol Geotherm Res 1996 73 141 155 10.1016/0377-0273(96)00021-2

[B78] Arnold EN Arribas O Carranza S Systematics of the Palaearctic and Oriental lizard tribe Lacertini (Squamata: Lacertidae: Lacertinae), with descriptions of eight new genera Zootaxa 2007 1430 1 86

[B79] Brown RP Yang Z Bayesian dating of shallow phylogenies with a relaxed clock Syst Biol 2010 59 119 10.1093/sysbio/syp082 20525625

[B80] Cox SC Carranza S Brown RP Divergence times and colonization of the Canary Islands by *Gallotia* lizards Mol Phylogenet Evol 2010 56 747 757 10.1016/j.ympev.2010.03.020 20307675

[B81] Maddison DR Maddison WP MacClade 4.0 2000 Sunderland, Massachusetts: Sinauer

[B82] Maddison WP Maddison DR Mesquite: A Modular System For Evolutionary Analysis. Version 2.73 http://mesquiteproject.org

[B83] Leviton AE Anderson SC Survey of the reptiles of the Sheikhdom of Abu Dhabi, Arabian Peninsula. Part II. Systematic account of the collection of reptiles made in the Sheikhdom of Abu Dhabi by John Gasperetti Proc Calif Acad Sci 1967 35 157 192

[B84] Haas G Two collections of Reptiles from Iraq, with descriptions of two new forms Copeia 1952 1952 20 22 10.2307/1437616

[B85] Murray JA Additions to the present knowledge of the vertebrate Zoology of Persia The Annals and Magazine of Natural History 1884 14 97 106

[B86] Werner F Allerlei aus dem Kriechtierleben im Käfig. II Zoologischer Garten, Frankfurt am Main 1899 40 12 24

[B87] Guichenot AA Histoire Naturelle Des Reptiles Et Des Poissons 1850 Paris: Imprimerie nationale

[B88] Gardner RAM Aeolianites and marine deposits of the Wahiba Sands: character and palaeoenvironments The Journal of Oman Studies 1988 3 1985 1987

[B89] Preusser F Radies D Driehorst F Matter A Late Quaternary history of the coastal Wahiba Sands, Sultanate of Oman J Quat Sci 2005 20 395 405 10.1002/jqs.922

[B90] Preusser F Radies D Matter A A 160,000-year record of Dune development and atmospheric circulation in Southern Arabia Science 2002 296 2018 2020 10.1126/science.1069875 12065834

[B91] Gallagher MD Arnold EN Reptiles and amphibians from the Wahiba Sands, Oman J Oman Stud, Spec Rep 1988 3 405 413

[B92] Blanford WT Descriptions of new lizards from Persia and Baluchistan Ann Mag Nat Hist 1874 13 453 455

[B93] Garzanti E Andò S Vezzoli G Dell'era D From rifted margins to foreland basins: investigating provenance and sediment dispersal across desert Arabia (Oman, U.A.E.) J Sediment Res 2003 73 572 588 10.1306/101702730572

[B94] Knowles LL Carstens BC Delimiting species without monophyletic gene trees Syst Biol 2007 56 887 895 10.1080/10635150701701091 18027282

[B95] Werner F Reptilien, Batrachier und Fische von Tripolis und Barka. Zoologische Jahrbucher Abteilung fur Systematik, Geographie und Biologie der Tiere 1909 27 595 646

[B96] Kratochvil L Frynta D Moravec J Third *Stenodactylus* in Africa: return of the forgotten form *Stenodactylus stenurus* Israel Journal of Zoology 2001 47 99 110 10.1560/MN76-08B0-HEFP-LJ4U

[B97] Barbour T Notes on some reptiles from Sinai and Syria Proceedings of the New England Zoological Club 1914 5 73 92

[B98] Baha El Din S A Guide to the Reptiles and Amphibians of Egypt 2006 Cairo and New York: The American University in Cairo Press, xvi

[B99] Loveridge A Revision of the African lizards of the family Gekkonidae Bulletin of the Mus Comp Zool, Harvard 1947 98 1 469

[B100] Loveridge A Checklist of the reptiles and amphibians of East Africa Bulletin of The Museum of Comparative Zoology 1957 117 151 362

[B101] Menzies MA Baker J Bosence D Dart C Davison I Hurford A Al'Kadasi M McClay K Nichols G Al'Subbary A Yelland A The timing of magmatism, uplift and crustal extension: preliminary observations from Yemen Geological Society, London, Special Publications 1992 68 293 304 10.1144/GSL.SP.1992.068.01.18

[B102] Autin J Leroy S Beslier MO DíAcremont E Razin P Ribodetti A Bellahsen N Robin C Al Toubi K Continental break up history of a deep magma poor margin based on seismic reflection data (northeastern Gulf of Aden margin, offshore Oman) Geophys J Int 2010 180 501 519 10.1111/j.1365-246X.2009.04424.x

[B103] Arnold EN Robinson MD Carranza S A preliminary analysis of phylogenetic relationships and biogeography of the dangerously venomous Carpet Vipers, *Echis* (Squamata, Serpentes, Viperidae) based on mitochondrial DNA sequences Amphibia-Reptilia 2009 30 273 282 10.1163/156853809788201090

[B104] Joger U Rocek Z Phylogenetic analysis of *Uromastyx *lizards, based on albumin immunological distances Studies in Herpetology 1986 Bonn, Germany: Societas Europaea Herpetologica 187 192

[B105] Girdler RW The Afro-Arabian rift system - an overview Tectonophysics 1991 197 139 153 10.1016/0040-1951(91)90038-T

[B106] Huang Y Clemens SC Liu W Wang Y Prell WL Large-scale hydrological change drove the late Miocene C4 plant expansion in the Himalayan foreland and Arabian Peninsula Geology 2007 35 531 534 10.1130/G23666A.1

[B107] Friend PF Rivers of the Lower Baynunah Formation, Emirate of Abu Dhabi, United Arab Emirates Fossil Vertebrates Of Arabia, With Emphasis On The Late Miocene Faunas, Geology, And Palaeoenvironments Of The Emirate Of Abu Dhabi, United Arab Emirates 1999 New Haven, Connecticut: Yale University Press 38 49

[B108] Zachos J Pagani M Sloan L Thomas E Billups K Trends, rhythms, and aberrations in global climate 65 Ma to Present Science 2001 292 686 693 10.1126/science.1059412 11326091

[B109] Paulo OS Dias C Bruford MW Jordan WC Nichols RA The persistence of Pliocene populations through the Pleistocene climatic cycles: evidence from the phylogeography of an Iberian lizard Proc R Soc London, Ser B 2001 268 1625 1630 10.1098/rspb.2001.1706 PMC1088786 11487410

[B110] Tzedakis PC Lawson IT Frogley MR Hewitt GM Preece RC Buffered tree population changes in a quaternary refugium: evolutionary implications Science 2002 297 2044 2047 10.1126/science.1073083 12242441

[B111] Bons J Geniez P Amphibians And Reptiles Of Morocco 1996 Barcelona: Asociación herpetológica Española

[B112] Schleich HH Kästle W Kabisch K Amphibians And Reptiles Of North Africa: Biology, Systematics, Field Guide 1996 Königstein, Germany: Koeltz Scientific Books

