# Supplementary material for: Conquering the Sahara and Arabian deserts: systematics and biogeography of Stenodactylus geckos (Reptilia: Gekkonidae)
Source: BMC Evol Biol. 2012 Dec 31;12:258. doi: 10.1186/1471-2148-12-258 (PMC3582542; doi:10.1186/1471-2148-12-258)
Supplement: Additional file 1: Table S1 — Information on the specimens used in the phylogenetic analyses. [file 1471-2148-12-258-S1.doc]

Table S1. **Information** **on** **the** **specimens** **used** **in** **the** **phylogenetic** **analyses**

| **Locality Code** | **Species** | **DNA Extraction Code** | **Voucher (or Tissue) Code** | **Country or Region** | **Locality** | **Genbank accession codes** |
| --- | --- | --- | --- | --- | --- | --- |
|  |  |  |  |  |  | **(12S/16S/c***-****mos*/*RAG-2*)** |
| 1 | *Stenodactylus affinis - 1* | M131 |  | Iran | Heled National Park | KC190679 / KC190873 / - / KC1901056 |
| 2 | *Stenodactylus affinis - 2* | M198 |  | Iran | Bandar e Khamir, semidesert | KC190677 / KC190871 / - / - |
| 3 | *Stenodactylus affinis - 3*** | M7 | BEV.10036 | Kuwait | 500 m W of Sulaibikhat Reserve, W. Kuwait City | KC190675 / KC190869 / KC190946 / KC191054 |
| 4 | *Stenodactylus affinis - 4* | M8 | BEV.10095 | Kuwait | Mina Said | KC190676 / KC190870 / KC190947 / KC191055 |
| 4 | *Stenodactylus affinis - 5* | M32 | BEV.10096 | Kuwait | Mina Said | KC190674 / KC190868 / KC190945 / KC191053 |
| 73 | *Stenodactylus affinis - 6* | M132 |  | Iran |  | - / KC190913 |
| 73 | *Stenodactylus affinis - 7* | M133 |  | Iran |  | KC190678 / KC190872 |
| 5 | *Stenodactylus cf. arabicus - 1*** | M10 |  | Oman | S of Al Mintrib | KC190696 / KC190890 / KC190998 / KC191116 |
| 5 | *Stenodactylus cf. arabicus - 2* | M36 |  | Oman | S of Al Mintrib | KC190697 / KC190891 / KC190999 / KC191117 |
| 6 | *Stenodactylus cf. arabicus - 3* | M166 |  | Oman | Al Mintrib, Sharqiya Sands | KC190699 / KC190893 / KC191000 / KC191118 |
| 7 | *Stenodactylus arabicus - 4* | M129 |  | Qatar | 15 km NE of As Salwa | KC190694 / KC190888 / KC190996 / KC191115 |
| 8 | *Stenodactylus arabicus - 5* | M9 |  | Oman | Sand dunes N of Shisur | KC190695 / KC190889 / KC190997 / KC191112 |
| 8 | *Stenodactylus arabicus - 6* | M33 |  | Oman | Sand dunes N of Shisur | KC190692 / KC190886 / KC191001 / KC191113 |
| 9 | *Stenodactylus arabicus - 7*** | M34 | BEV.10042 | Kuwait | Wafrah Farms, 20 km E of Wafrah | KC190690 / KC190884 / KC190994 / KC191110 |
| 9 | *Stenodactylus arabicus - 8* | M35 | BEV.10043 | Kuwait | Wafrah Farms, 20 km E of Wafrah | KC190691 / KC190885 / KC190995 / KC191111 |
| 10 | *Stenodactylus arabicus - 9* | E150536 |  | UAE | Dhafra Beach Hotel | KC190693 / KC190887 / KC191011 / KC191114 |
| 6 | *Stenodactylus cf. arabicus - 10* | M167 |  | Oman | Al Mintrib, Sharqiya Sands | KC190698 / KC190892 |
| 11 | *Stenodactylus doriae - 1* | M31 |  | Oman | Al-Areesh Desert Camp, Sharqiya Sands | KC190643 / KC190837 / KC190936 / KC191044 |
| 6 | *Stenodactylus doriae - 2* | M169 |  | Oman | Al Mintrib, Sharqiya Sands | KC190644 / KC190838 / KC190937 / KC191121 |
| 12 | *Stenodactylus doriae - 3*** | E150538 |  | UAE | Sand dunes at Al Ain | KC190653 / KC190847 / KC190939 / KC191050 |
| 13 | *Stenodactylus doriae - 4* | M185 | BEV.T3759 | Jordan | Between Aqaba and Wadi Rum | KC190650 / KC190844 / KC190941 / KC191047 |
| 14 | *Stenodactylus doriae - 5* | M28 | BEV.10037 | Kuwait | 13 km N-NE of Jahra | KC190646 / KC190840 / KC190938 / KC191045 |
| 15 | *Stenodactylus doriae - 6* | M30 | BEV.8483 | Israel | 14 km N of Eilot | KC190649 / KC190843 / KC190940 / KC191046 |
| 6 | *Stenodactylus doriae - 7* | M170 |  | Oman | Al Mintrib, Sharqiya Sands | KC190645 / KC190839 |
| 74 | *Stenodactylus doriae - 8* | M12 |  | Oman | Al-Areesh Desert Camp, Sharqiya Sands | KC190642 / KC190836 |
| 75 | *Stenodactylus doriae - 9* | E150537 |  | UAE | Al Aya, Abu Dhabi | KC190652 / KC190846 |
| 18 | *Stenodactylus doriae - 10* | M11 | BEV.10038 | Kuwait | Wafrah Farms, 20 km E of Wafrah | KC190647 / KC190841 |
| 13 | *Stenodactylus doriae - 11* | M186 | BEV.T3760 | Jordan | Between Aqaba and Wadi Rum | KC190651 / KC190845 |
| 15 | *Stenodactylus doriae - 12* | M29 | BEV.8482 | Israel | 14 km N of Eilot | KC190648 / KC190842 |
| 76 | *Stenodactylus doriae - 13* | M120 |  | Qatar |  | KC190654 / KC190848 |
| 136 | *Stenodactylus doriae - 14* | M210 |  | Yemen | Ma'rib | KC190720 / KC190922 / KC191030 / KC191138 |
| 136 | *Stenodactylus doriae - 15* | M211 |  | Yemen | Ma'rib | KC190721 / KC190923 / KC191031 / KC191139 |
| 136 | *Stenodactylus doriae - 16* | M212 |  | Yemen | Ma'rib | KC190722 / KC190924 |
| 16 | *Stenodactylus grandiceps - 1*** | M13 |  | Jordan | Wadi Bayir | KC190668 / KC190862 / KC190952 / KC191062 |
| 16 | *Stenodactylus grandiceps - 2* | M14 |  | Jordan | Wadi Bayir | KC190667 / KC190861 / KC190951 / KC191061 |
| 17 | *Stenodactylus grandiceps - 3* | M191 | BEV.10911 | Jordan | 6 km E of Shawbak | KC190673 / KC190867 / KC190953 / KC191063 |
| 77 | *Stenodactylus grandiceps - 4* | M158 | NHMC80.3.107.6 | Jordan | 4km N of Al Manshiyya | KC190669 / KC190863 |
| 77 | *Stenodactylus grandiceps - 5* | M159 | NHMC80.3.107.2 | Jordan | 4km N of Al Manshiyya | KC190670 / KC190864 |
| 17 | *Stenodactylus grandiceps - 6* | M189 | BEV.10909 | Jordan | 6 km E of Shawbak | KC190671 / KC190865 |
| 17 | *Stenodactylus grandiceps - 7* | M190 | BEV.10910 | Jordan | 6 km E of Shawbak | KC190672 / KC190866 |
| 78 | *Stenodactylus grandiceps - 8* | M160 | NHMC80.3.107.1 | Jordan | 30 km W of Azraq | - / KC190912 |
| 19 | *Stenodactylus grandiceps - 9* | M203 |  | Jordan | Azraq | KC190714 / KC190915 / KC191027 / KC191135 |
| 19 | *Stenodactylus grandiceps - 10* | M204 |  | Jordan | Azraq | KC190715 / KC190916 / KC191028 / KC191136 |
| 19 | *Stenodactylus grandiceps - 11* | M205 |  | Jordan | Azraq | KC190716 / KC190917 |
| 20 | *Stenodactylus leptocosymbotes - 1* | M18 |  | Oman | Thumrait | KC190662 / KC190856 / KC190943 / KC191052 |
| 21 | *Stenodactylus leptocosymbotes - 2* | M23 |  | Oman | 4km SW Al Maaymir | KC190655 / KC190849 / KC191012 / KC191051 |
| 22 | *Stenodactylus leptocosymbotes - 3* | M25 |  | Oman | 2.5 km SE Ar Rumayliyah | KC190657 / KC190851 / KC190942 / KC191048 |
| 23 | *Stenodactylus leptocosymbotes - 4*** | M26 |  | Oman | Wadi Maahdi, Masirah island | KC190666 / KC190860 / KC190944 / KC191049 |
| 24 | *Stenodactylus leptocosymbotes - 5* | E1505313 |  | UAE | Al Ain | KC190664 / KC190858 / KC191013 / KC191105 |
| 20 | *Stenodactylus leptocosymbotes - 6* | e903611 |  | Oman | Thumrait | KC190660 / KC190854 |
| 20 | *Stenodactylus leptocosymbotes - 7* | e903612 |  | Oman | Thumrait | KC190661 / KC190855 |
| 79 | *Stenodactylus leptocosymbotes - 8* | M24 |  | Oman |  | KC190658 / KC190852 |
| 21 | *Stenodactylus leptocosymbotes - 9* | e90361 |  | Oman |  | KC190656 / KC190850 |
| 21 | *Stenodactylus leptocosymbotes - 10* | e90363 |  | Oman |  | KC190659 / KC190853 |
| 24 | *Stenodactylus leptocosymbotes - 11* | e90366 |  | UAE | Al Ain | KC190663 / KC190857 |
| 24 | *Stenodactylus leptocosymbotes - 12* | e90368 |  | UAE | Al Ain | KC190665 / KC190859 |
| 25 | *Stenodactylus mauritanicus - 1* | E1505339 |  | Egypt | El Omayed protected area | KC190577 / - / KC191018 / KC191083 |
| 26 | *Stenodactylus mauritanicus - 2* | E1505353 |  | Tunisia | Oued shili, 31 km NE of Tozeur | KC190566 / KC190770 / KC191016 / KC191077 |
| 27 | *Stenodactylus mauritanicus - 3* | M111 |  | Tunisia | Crossroad to Jbel Chambi | KC190567 / KC190771 / KC190976 / KC191108 |
| 28 | *Stenodactylus mauritanicus - 4* | M112 |  | Tunisia | Jbel Tamesmida | KC190568 / KC190772 / KC190977 / KC191078 |
| 29 | *Stenodactylus mauritanicus - 5*** | M157 | NHMC80.3.152.1 | Libya | 30 km S of Misirata | KC190579 / KC190782 / KC190981 / KC191074 |
| 30 | *Stenodactylus mauritanicus - 6*** | M80 |  | Morocco | 44 km S-SW of Sidi Ifni | KC190590 / KC190791 / KC190978 / KC191072 |
| 31 | *Stenodactylus mauritanicus - 7* | M88 |  | W. Sahara, Morocco | 84 km N of Boudjour | KC190595 / KC190796 / KC191017 / KC191085 |
| 31 | *Stenodactylus mauritanicus - 8* | M110 |  | W. Sahara, Morocco | 5 km NE of Lemsid, | KC190596 / KC190797 / KC191008 / KC191086 |
| 32 | *Stenodactylus mauritanicus - 9* | M115 |  | W. Sahara, Morocco | 100 km S of Boujdour | KC190597 / KC190798 / KC191009 / KC191079 |
| 33 | *Stenodactylus mauritanicus - 10* | M176 | BEV.10835 | W. Sahara, Morocco | Oued Lcraa, 170 km S-SW of Boujdour | KC190599 / KC190800 / KC190980 / KC191080 |
| 34 | *Stenodactylus mauritanicus - 11* | M177 | BEV.10836 | Morocco | 12 km S-SW Tan Tan | KC190587 / KC190789 / KC190979 / KC191073 |
| 80 | *Stenodactylus mauritanicus - 12* | M55 | BEV.9157 | Mauritania | Cap Blanc | KC190600 / KC190801 |
| 32 | *Stenodactylus mauritanicus - 13* | M116 |  | W. Sahara, Morocco | 100 km S of Boujdour | KC190598 / KC190799 |
| 43 | *Stenodactylus mauritanicus - 14* | M74 |  | W. Sahara, Morocco | 25 km W of Smara | KC190583 / KC190786 |
| 43 | *Stenodactylus mauritanicus - 15* | M75 |  | W. Sahara, Morocco | 25 km W of Smara | KC190584 / KC190787 |
| 81 | *Stenodactylus mauritanicus - 16* | M49 | BEV.2387 | Morocco | Between Khnifiss and Tantan | KC190588 / - |
| 81 | *Stenodactylus mauritanicus - 17* | M50 | BEV.2388 | Morocco | Between Khnifiss and Tantan | KC190586 / - |
| 82 | *Stenodactylus mauritanicus - 18* | M54 | BEV.7889 | Morocco | 4 km Nwof Tantan | KC190585 / KC190788 |
| 83 | *Stenodactylus mauritanicus - 19* | M82 |  | Morocco | S of Sidi Ifni | KC190592 / KC190793 |
| 84 | *Stenodactylus mauritanicus - 20* | M83 |  | Morocco | S of Sidi Ifni | KC190593 / KC190794 |
| 85 | *Stenodactylus mauritanicus - 21* | M81 |  | Morocco | S of Sidi Ifni | KC190591 / KC190792 |
| 86 | *Stenodactylus mauritanicus - 22* | M84 |  | Morocco | S of Sidi Ifni | KC190594 / KC190795 |
| 87 | *Stenodactylus mauritanicus - 23* | M79 |  | Morocco | S of Sidi Ifni | KC190589 / KC190790 |
| 88 | *Stenodactylus mauritanicus - 24* | M142 | NHMC80.3.88.40 | Morocco | 146 km E of Ouarzazate | KC190602 / KC190803 |
| 89 | *Stenodactylus mauritanicus - 25* | M137 | NHMC80.3.88.45 | Libya | 55 km S of Misratah | KC190578 / KC190781 |
| 90 | *Stenodactylus mauritanicus - 26* | M153 | NHMC80.3.88.1 | Libya | Libya - Egypt borders | - / KC190911 |
| 91 | *Stenodactylus mauritanicus - 27* | M135 | NHMC80.3.88.47 | Libya | Qaminis, 120 km NE of Adjedabia | KC190571 / KC190775 |
| 91 | *Stenodactylus mauritanicus - 28* | M154 | NHMC80.3.153.2 | Libya | Qaminis, 120 km NE of Adjedabia | KC190572 / KC190776 |
| 91 | *Stenodactylus mauritanicus - 29* | M155 | NHMC80.3.153.1 | Libya | Qaminis, 120 km NE of Adjedabia | KC190575 / KC190779 |
| 92 | *Stenodactylus mauritanicus - 30* | M141 | NHMC80.3.88.41 | Morocco | 30 km NE of Goulmina | KC190601 / KC190802 / KC190988 / KC191075 |
| 93 | *Stenodactylus mauritanicus - 31* | M140 | NHMC80.3.88.42 | Libya | Kikla | KC190580 / KC190783 |
| 94 | *Stenodactylus mauritanicus - 32* | M156 | NHMC80.3.152.2 | Libya | Ain Tagnit | KC190581 / KC190784 |
| 95 | *Stenodactylus mauritanicus - 33* | M151 | NHMC80.3.88.29 | Libya | 75 km W of Tobruk | - / KC190910 |
| 96 | *Stenodactylus mauritanicus - 34* | M134 | NHMC80.3.88.9 | Libya | Igdeida semidesert | KC190574 / KC190778 |
| 96 | *Stenodactylus mauritanicus - 35* | M152 | NHMC80.3.88.28 | Libya | Igdeida semidesert | KC190573 / KC190777 |
| 97 | *Stenodactylus mauritanicus - 36* | M138 | NHMC80.3.88.44 | Libya | Al Mabne | KC190576 / KC190780 |
| 98 | *Stenodactylus mauritanicus - 37* | E1505349 |  | Morocco | 17 Km Nortwest of Missour | KC190558 / - |
| 99 | *Stenodactylus mauritanicus - 38* | M56 | BEV.9985 | Morocco | Al Baten plain | KC190564 / - |
| 99 | *Stenodactylus mauritanicus - 39* | M57 | BEV.9986 | Morocco | Al Baten plain | KC190561 / - |
| 99 | *Stenodactylus mauritanicus - 40* | M58 | BEV.9990 | Morocco | Al Baten plain | KC190560 / - |
| 100 | *Stenodactylus mauritanicus - 41* | M98 | MCCR1160-1 | Morocco | Between Debdou and Ain Benimathar | KC190562 / KC190769 |
| 101 | *Stenodactylus mauritanicus - 42* | M103 |  | Morocco | N of Msoun, Taza | KC190565 / - |
| 101 | *Stenodactylus mauritanicus - 43* | M76 |  | Morocco | N of Msoun, Taza | KC190559 / KC190768 |
| 102 | *Stenodactylus mauritanicus - 44* | E1505350 |  | Morocco | 19 Km W of El Aioum | KC190563 / - |
| 103 | *Stenodactylus mauritanicus - 45* | M148 | NHMC80.3.88.34 | Tunisia | 2 km N of Ouled Monaceur | - / KC190909 |
| 28 | *Stenodactylus mauritanicus - 46* | M113 |  | Tunisia | Jbel Tamesmida, base of | KC190596 / KC190773 |
| 28 | *Stenodactylus mauritanicus - 47* | M114 |  | Tunisia | Jbel Tamesmida, base of | KC190570 / KC190774 |
| 104 | *Stenodactylus mauritanicus - 48* | M124 |  | Libya |  | KC190582 / KC190785 |
| 35 | *Stenodactylus petrii - 1*** | M4 | BEV.8987 | Egypt | Wadi El Natrun | KC190603 / KC190804 / KC190954 / KC191093 |
| 36 | *Stenodactylus petrii - 2* | E1505316 |  | Egypt | E of Al Arish, Sinai | KC190610 / KC190811 / KC190955 / KC191094 |
| 37 | *Stenodactylus petrii - 3* | M165 | HUJR-23776 | Israel | NW Negev sands, Ovitz field Haluzza | KC190611 / KC190812 / KC190956 / KC191107 |
| 38 | *Stenodactylus petrii - 4* | M6 | BEV.9131 | Mauritania | El Beyed | KC190612 / KC190813 / KC190957 / KC191102 |
| 39 | *Stenodactylus petrii - 5* | M78 |  | Morocco | Erg Chebbi | KC190617 / KC190818 / KC190960 / KC191095 |
| 40 | *Stenodactylus petrii - 6* | M105 | BEV.10157 | Algeria | Tassili N'Ajer | KC190613 / KC190814 / KC190958 / KC191106 |
| 41 | *Stenodactylus petrii - 7* | M107 | BEV.10181 | Algeria | Tisras | KC190619 / KC190819 / KC190959 / - |
| 42 | *Stenodactylus petrii - 8* | M119 |  | Mauritania | Tarf Tazazmout | KC190615 / KC190816 / KC191007 / KC191133 |
| 43 | *Stenodactylus petrii - 9*** | M2 |  | W. Sahara, Morocco | 25 km W of Smara | KC190626 / KC190826 / KC190964 / KC191103 |
| 44 | *Stenodactylus petrii - 10* | E1505326 |  | Mauritania | Oued Choum, Adrar | KC190631 / KC190829 / KC191019 / KC191096 |
| 45 | *Stenodactylus petrii - 11* | M178 | BEV.T3692 | W. Sahara, Morocco | 90 km NW of Aousserd | KC190621 / KC190821 / KC190965 / KC191097 |
| 46 | *Stenodactylus petrii - 12* | M180 | BEV.10842 | W. Sahara, Morocco | 22 km NW of Aousserd | KC190623 / KC190823 / KC190966 / KC191098 |
| 46 | *Stenodactylus petrii - 13* | M182 | BEV.10844 | W. Sahara, Morocco | 22 km NW of Aousserd | KC190625 / KC190825 / KC190967 / KC191099 |
| 105 | *Stenodactylus petrii - 14* | E1505321 |  | Mauritania | 35 Km S of Bennichchab | KC190632 / - |
| 105 | *Stenodactylus petrii - 15* | E1505322 |  | Mauritania | 35 Km S of Bennichchab | KC190633 / - |
| 105 | *Stenodactylus petrii - 16* | E1505323 |  | Mauritania | 35 Km S of Bennichchab | KC190629 / - |
| 106 | *Stenodactylus petrii - 17* | E1505324 |  | Mauritania | Ben Amira (Adrar) | KC190620 / KC190820 |
| 107 | *Stenodactylus petrii - 18* | E1505329 |  | Mauritania | 20 Km W of Tmeimichat | KC190628 / KC190828 |
| 44 | *Stenodactylus petrii - 19* | E1505325 |  | Mauritania | Oued Choum (Adrar) | KC190630 / - |
| 44 | *Stenodactylus petrii - 20* | E1505327 |  | Mauritania | Oued Choum (Adrar) | KC190627 / KC190827 |
| 46 | *Stenodactylus petrii - 21* | M179 | BEV.10841 | W. Sahara, Morocco | 22 km NW of Aousserd | KC190622 / KC190822 |
| 46 | *Stenodactylus petrii - 22* | M181 | BEV.10843 | W. Sahara, Morocco | 22 km NW of Aousserd | KC190624 / KC190824 |
| 108 | *Stenodactylus petrii - 23* | E1505319 |  | Mauritania | 30 Km N of Zouerat | KC190634 / - |
| 109 | *Stenodactylus petrii - 24* | M106 | BEV.10180 | Algeria |  | KC190614 / KC190815 |
| 110 | *Stenodactylus petrii - 25* | E1505328 |  | W. Sahara, Morocco | Tiffarity | KC190916 / KC190817 |
| 111 | *Stenodactylus petrii - 26* | M93 | MCC1481 | Egypt | N Sinai, Zaranik National Park | KC190608 / KC190809 |
| 35 | *Stenodactylus petrii - 27* | M3 | BEV.8986 | Egypt | Wadi El Natrun | KC190607 / KC190808 |
| 35 | *Stenodactylus petrii - 28* | M5 | BEV.8988 | Egypt | Wadi El Natrun | KC190604 / KC190805 |
| 25 | *Stenodactylus petrii - 29* | E1505317 |  | Egypt | El Omayed protected area | KC190606 / KC190807 |
| 112 | *Stenodactylus petrii - 30* | M94 | MCC1480-1 | Egypt | N Sinai, near Bir el Abd | KC190609 / KC190810 |
| 113 | *Stenodactylus petrii - 31* | E1505318 |  | Egypt | N Sinai, Zaranik National Park | KC190605 / KC190806 |
| 114 | *Stenodactylus petrii - 32* | M102 |  | Morocco | 15 Km S of Erfoud | KC190618 / - |
| 53 | *Stenodactylus petrii - 33* | M92 | MCC1329 | Tunisia | 6 km W of Nefta | KC190636 / KC190830 / KC190961 / KC191104 |
| 54 | *Stenodactylus petrii - 34* | E1505331 |  | Tunisia | 6 km W of Nefta | KC190639 / KC190833 / KC190962 / KC191100 |
| 54 | *Stenodactylus petrii - 35* | E1505332 |  | Tunisia | 6 km W of Nefta | KC190637 / KC190831 / KC190963 / KC191101 |
| 117 | *Stenodactylus petrii - 36* | M97 | MCC1335 | Tunisia | 34 km S of Hazoua | KC190638 / KC190832 |
| 54 | *Stenodactylus petrii - 37* | M1 |  | Tunisia | 6 Km west of Nefta | KC190635 / - |
| 47 | *Stenodactylus pulcher - 1*** | M122 |  | Yemen | Mukkalla | KC190700 / KC190894 / KC191002 / KC191119 |
| 48 | *Stenodactylus pulcher - 2* | M130 |  | Yemen | Al Rayan | KC190701 / KC190895 / KC191003 / KC191120 |
| 49 | *Stenodactylus slevini - 1*** | M19 |  | Jordan | Abar al Hazim | KC190680 / KC190874 / KC190950 / KC191057 |
| 13 | *Stenodactylus slevini - 2* | M184 | BEV.10884 | Jordan | Between Aqaba and Wadi Rum | KC190682 / KC190876 / KC190948 / KC191132 |
| 13 | *Stenodactylus slevini - 3* | M187 |  | Jordan | Between Aqaba and Wadi Rum | KC190683 / KC190877 / KC190949 / KC191058 |
| 50 | *Stenodactylus slevini - 4* | M20 | BEV.10065 | Kuwait | Sabah Al-Ahmed Natural Reserve | KC190685 / KC190879 / KC191014 / KC191059 |
| 51 | *Stenodactylus slevini - 5* | M127 |  | Qatar | 26 km SE of As Salwa | KC190689 / KC190883 / - / - |
| 52 | *Stenodactylus slevini - 6* | E1505334 |  | UAE | Tabal Dani | KC190687 / KC190881 / KC190993 / KC191060 |
| 115 | *Stenodactylus slevini - 7* | E1505335 |  | UAE | Tabal Dannah (UAE) | KC190688 / KC190882 |
| 13 | *Stenodactylus slevini - 8* | M183 | BEV.10883 | Jordan | Between Aqaba and Wadi Rum | KC190681 / KC190875 |
| 13 | *Stenodactylus slevini - 9* | M188 | BEV.T3762 | Jordan | Between Aqaba and Wadi Rum | KC190684 / KC190878 |
| 116 | *Stenodactylus slevini - 10* | M38 | BEV.T1501 | Kuwait | Ratqa, Kuwait-Irak borders | KC190686 / KC190880 |
| 137 | *Stenodactylus stenurus*** | M199 | CUP\REPT\LIB\143 | Tunisia | Chaffar | KC190713 / KC190914 / KC191026 / KC191134 |
| 25 | *Stenodactylus sthenodactylus - 1*** | M22 |  | Egypt | El Omayed protected area | KC190552 / KC190762 / KC190990 / KC191070 |
| 55 | *Stenodactylus sthenodactylus - 2* | M64 | BEV.7219 | Egypt | 10 km N of Hurghada, El Gouna | KC190524 / KC190737 / KC191015 / - |
| 56 | *Stenodactylus sthenodactylus - 3* | M70 | BEV.8990 | Egypt | Wadi Gharandal, Sinai | KC190526 / KC190739 / KC190968 / KC191066 |
| 57 | *Stenodactylus sthenodactylus - 4* | M73 | BEV.9027 | Egypt | 26 km S-SW of Beer Abraq | KC190543 / KC190754 / KC190971 / KC191067 |
| 58 | *Stenodactylus sthenodactylus - 5* | M123 |  | Egypt | Farafra Oasis | KC190550 / KC190760 / KC191010 / KC191068 |
| 59 | *Stenodactylus sthenodactylus - 6* | M172 | BEV.10370 | Egypt | Abu Simbel | KC190548 / KC190758 / KC190972 / KC191069 |
| 37 | *Stenodactylus sthenodactylus - 7* | M161 | HUJR-23793 | Israel | NW Negev sands, Ovitz field Haluzza | KC190522 / KC190735 / KC190989 / KC191076 |
| 60 | *Stenodactylus sthenodactylus - 8* | M171 | BEV.10199 | Israel | 5 km S-SW of Boker, Negev | KC190529 / KC190742 / KC190982 / KC191084 |
| 61 | *Stenodactylus sthenodactylus - 9* | M192 | BEV.T3951 | Jordan | 8 km of Ad-Dura,Saudi Arabia borders | KC190555 / KC190765 / KC190983 / KC191071 |
| 63 | *Stenodactylus sthenodactylus - 11* | M136 | NHMC80.3.88.46 | Libya | 20 km W of Derj oasis | KC190541 / KC190752 / KC190973 / KC191081 |
| 64 | *Stenodactylus sthenodactylus - 12* | M193 | BEV.T4135 | Algeria | Oued Dider, Aguelmane Assar | KC190537 / KC190749 / KC190969 / KC191082 |
| 65 | *Stenodactylus sthenodactylus - 13* | M117 |  | Mauritania | 18 km NW of Baie d'Arguin | KC190540 / KC190751 / KC190975 / KC191090 |
| 66 | *Stenodactylus sthenodactylus - 14* | M118 |  | Mauritania | 5 km S of Tîgjafât | KC190534 / KC190747 / KC190974 / KC191109 |
| 67 | *Stenodactylus sthenodactylus - 15* | M173 | BEV.10832 | W. Sahara, Morocco | 118 km NW of Aousserd | KC190554 / KC190764 / KC190987 / KC191091 |
| 68 | *Stenodactylus sthenodactylus - 16* | M174 | BEV.10833 | W. Sahara, Morocco | 42 km NW of Aousserd | KC190533 / KC190746 / KC190986 / KC191089 |
| 69 | *Stenodactylus sthenodactylus - 17*** | M175 | BEV.10834 | W. Sahara, Morocco | 59 km NW of Aousserd | KC190531 / KC190744 / KC190985 / KC191088 |
| 70 | *Stenodactylus sthenodactylus - 18* | M87 |  | Mauritania | 170 km E of Bou Lenoir | KC190532 / KC190745 / KC190984 / KC191087 |
| 118 | *Stenodactylus sthenodactylus - 19* | M60 | BEV.2410 | Mauritania | 26 km S of Chott Boul | KC190538 / - |
| 119 | *Stenodactylus sthenodactylus - 20* | M53 | BEV.2411 | Mauritania | 70 km E-NE Akjoujt | KC190530 / KC190743 |
| 70 | *Stenodactylus sthenodactylus - 21* | M109 |  | Mauritania | Inêl, 5km S of Dakhlet-Nouâdhibou | - / KC190906 |
| 120 | *Stenodactylus sthenodactylus - 22* | M86 |  | Mauritania |  | KC190539 / KC190750 |
| 120 | *Stenodactylus sthenodactylus - 23* | M108 |  | Mauritania | Boû Lanouâr, 17km E of Dakhlet-Nouâdhibou | - / KC190907 |
| 121 | *Stenodactylus sthenodactylus - 24* | E1505336 |  | Egypt | N of Gebel Elba | KC190542 / KC190753 |
| 122 | *Stenodactylus sthenodactylus - 25* | M72 | BEV.9004 | Egypt | 7 km NE of Abu Simbel | KC190549 / KC190759 |
| 123 | *Stenodactylus sthenodactylus - 26* | M66 | BEV.7222 | Egypt | Abu Simbel | KC190546 / KC190756 |
| 123 | *Stenodactylus sthenodactylus - 27* | M68 | BEV.7242 | Egypt | Abu Simbel | KC190547 / KC190757 |
| 124 | *Stenodactylus sthenodactylus - 28* | E1505345 |  | Egypt | Red Sea Coast, Egypt | KC190544 / - |
| 125 | *Stenodactylus sthenodactylus - 29* | E1505347 |  | Egypt | Wadi ‘Adb al Malik | KC190545 / KC190755 |
| 55 | *Stenodactylus sthenodactylus - 30* | M65 | BEV.7220 | Egypt | 10 km N of Hurghada, El Gouna | KC190525 / KC190738 |
| 126 | *Stenodactylus sthenodactylus - 31* | M62 | BEV.7215 | Egypt | Feiran Oasis, Sinai | KC190528 / KC190741 |
| 56 | *Stenodactylus sthenodactylus - 32* | M71 | BEV.8991 | Egypt | Wadi Gharandal, Sinai | KC190527 / KC190740 |
| 127 | *Stenodactylus sthenodactylus - 33* | E1505346 |  | Egypt | Siwa Oasis | KC190536/ - |
| 128 | *Stenodactylus sthenodactylus - 34* | M146 | NHMC80.3.88.37 | Egypt | Wadi Sudr, 10 km SE of Qa'lat el Jundi | - / KC190908 |
| 129 | *Stenodactylus sthenodactylus - 35* | M63 | BEV.7216 | Egypt | 10 km N of Aïn Sukhna | KC190519 / KC190732 |
| 130 | *Stenodactylus sthenodactylus - 36* | M61 | BEV.7214 | Egypt | 25 km W of Suez | KC190517 / KC190730 |
| 130 | *Stenodactylus sthenodactylus - 37* | M67 | BEV.7241 | Egypt | 25 km W of Suez | KC190518 / KC190731 |
| 111 | *Stenodactylus sthenodactylus - 38* | M90 | MCC1479 | Egypt | N Sinai, Zaranik National Park | KC190521 / KC190734 |
| 131 | *Stenodactylus sthenodactylus - 39* | M89 | MCC1449 | Libya | Ghadames | KC190535 / KC190748 |
| 132 | *Stenodactylus sthenodactylus - 40* | E1505337 |  | Egypt | 30 km NE of Cairo | KC190516 / KC190729 |
| 133 | *Stenodactylus sthenodactylus - 41* | M69 | BEV.8989 | Egypt | Wadi El Natrun | KC190520 / KC190733 |
| 134 | *Stenodactylus sthenodactylus - 42* | M125 |  | Egypt |  | KC190523 / KC190736 |
| 25 | *Stenodactylus sthenodactylus - 43* | E1505340 |  | Egypt | El Omayed protected area | KC190553 / KC190763 |
| 25 | *Stenodactylus sthenodactylus - 44* | E1505342 |  | Egypt | El Omayed protected area | KC190551 / KC190761 |
| 62 | *Stenodactylus sth. zavattarii - 1* | M101 | MCCR1372 | Kenya | Between Gatab and South Horr, Samburu distr. | KC190557 / KC190767 / KC190970 / KC191092 |
| 135 | *Stenodactylus sth. zavattarii - 2* | M95 | MCC1297-1 | Kenya | North Horr | KC190556 / KC190766 |
| 71 | *Stenodactylus yemenensis - 1*** | M126 |  | Yemen | Mokka | KC190640 / KC190834 / KC190991 / KC191064 |
| 72 | *Stenodactylus yemenensis - 2* | M128 |  | Yemen | N. Yukhtal | KC190641 / KC190835 / KC190992 / KC191065 |
| 138 | *Stenodactylus yemenensis - 3* | M206 |  | Yemen | N of Aden | KC190717 / KC190918 / KC191029 / KC191137 |
| 138 | *Stenodactylus yemenensis - 4* | M207 |  | Yemen | N of Aden | KC190718 / KC190919 |
| 138 | *Stenodactylus yemenensis - 5* | M208 |  | Yemen | N of Aden | KC190719 / KC190920 |
| 138 | *Stenodactylus yemenensis - 6* | M209 |  | Yemen | N of Aden | - / KC190921 |
| 139 | *Stenodactylus yemenensis - 7* | M213 |  | Yemen | N of Lahj, Wadi Tuban | KC190723 / KC190925 / KC191032 / KC191140 |
| 139 | *Stenodactylus yemenensis - 8* | M214 |  | Yemen | N of Lahj, Wadi Tuban | KC190724 / KC190926 |
|  | **Outgroups** |  |  |  |  |  |
|  | *Agamura persica*** | E18011017 |  | Iran |  | DQ852726 / - / KC191025 / - |
|  | *Aristelliger georgeensis** | E280511 |  | Belize |  | KC190934 / KC190927 / KC191043 / KC191141 |
|  | *Bunopus tuberculatus*** | Buntub |  | Kuwait |  | EU589160 / - / AF148706 / - |
|  | *Crossobamon orientalis*** | Croori |  | India |  | HM921159 / HM040944 / DQ852730 / - |
|  | *Euleptes europaea** | E260671 |  | Italy |  | KC190935 / - / KC191042 / KC191142 |
|  | *Gekko gecko** | Gekgec |  | China/Indonesia |  | NC007627 / NC007627 / EF534939 / EF534981 |
|  | *Gekko vittatus** | Gekvit |  |  |  | NC008772 / NC008772 / - / - |
|  | *Hemidactylus frenatus*** | Hemfre |  | Indonesia |  | GQ245970 / GQ245970 / - / EF534982 |
|  | *Pseudoceramodactylus khobarensis - 1*** | M16 | BEV.10039 | Kuwait | Wafrah Farms, 20 km E of Wafrah | KC190703 / KC190897 / KC191005 / KC191123 |
|  | *Pseudoceramodactylus khobarensis - 2* | M37 | BEV.10040 | Kuwait | Wafrah Farms, 20 km E of Wafrah | KC190702 / KC190896 / KC191004 / KC191122 |
|  | *Pseudoceramodactylus khobarensis - 3* | M196 |  | Oman | Barr Al-Hickman | KC190704 / KC190898 / KC191006 / KC191124 |
|  | *Saurodactylus brosseti** | Saubro |  | Morocco |  | EU014300 / EF564006 / EF534928 / EF534970 |
|  | *Tarentola mauritanica** | Tarmau |  | Spain/Egypt |  | NC012366 / NC012366 / EU293686 / EU293731 |
|  | *Tarentola delalandii** | MtardeT |  | Canary Islands | Tenerife | AF186131 / KC190928 / KC191033 / KC191143 |
|  | *Tarentola delalandii** | MtardeP |  | Canary Islands | La Palma | AF186130 / KC190929 / KC191034 / KC191144 |
|  | *Tarentola boettgeri boettgeri** | E1011311 |  | Canary Islands | Gran Canaria | AF186125 / KC190930 / KC191035 / KC191145 |
|  | *Tarentola boettgeri bischoffi** | E101138 |  | Madeira | Selvagens | AF186128 / - / - / - |
|  | *Tarentola boettgeri hierrensis** | E1011310 |  | Canary Islands | El Hierro | KC190725 / - / KC191036 / KC191146 |
|  | *Tarentola boettgeri boettgeri** | E1011313 |  | Canary Islands | Gran Canaria | AF186123 / - / KC191037 / KC191147 |
|  | *Tarentola boettgeri boettgeri** | E1011314 |  | Canary Islands | Gran Canaria | AF186124 / - / KC191038 / KC191148 |
|  | *Teratoscincus scincus** | Mter1 | JFBM14252 | Turkmenistan |  | KC190726 / KC190931 / KC191039 / KC191149 |
|  | *Teratoscincus roborowskii** | Mter2 |  | China |  | KC190727 / KC190932 / KC191040 / KC191150 |
|  | *Teratoscincus microlepis** | Mter3 |  | Pakistan |  | KC190728 / KC190933 / KC191041 / KC191151 |
|  | *Tropiocolotes algericus - 1*** | MT5 | BEV.10862 | W. Sahara, Morocco | 175 km NW of Aousserd | KC190709 / KC190902 / - / - |
|  | *Tropiocolotes algericus - 2*** | MT6 | BEV.10860 | W. Sahara, Morocco | 110 km NW of Boudjour | KC190710 / KC190903 / KC191023 / KC191129 |
|  | *Tropiocolotes tripolitanus - 1*** | MT7 | BEV.10863 | W. Sahara, Morocco | Aousserd | KC190711 / KC190904 / KC191024 / KC191130 |
|  | *Tropiocolotes tripolitanus - 2*** | MT8 | BEV.9025 | Egypt | Wadi El Natrun | KC190712 / KC190905 / - / KC190031 |
|  | *Tropiocolotes nubicus*** | MT9 | BEV.9020 | Egypt | Abu Simbel | KC190706 / KC190900 / KC191020 / KC191125 |
|  | *Tropiocolotes steudneri*** | MT10 | BEV.10367 | Egypt | 175 km S of Safaga | KC190705 / KC190899 / KC191021 / KC191126 |
|  | *Tropiocolotes nattereri*** | MT11 | BEV.10886 | Jordan | Wadi Rum | KC190708 / KC190901 / - / KC190028 |
|  | *Tropiocolotes scorteccii*** | MT12 |  | Oman | Coast, 250 km E of Salalah | KC190707 / - / KC191022 / KC191127 |

Specimens are listed in alphabetical order, with the corresponding GenBank accession numbers. Specimens indicated with an asterisk (*) were included in the divergence time analysis, specimens indicated with a double asterisk (**) were included both in the phylogenetic and divergence time analyses, and specimens with no asterisk were included in the phylogenetic analysefs only. Locality codes refer to Figure 1. Voucher codes of specimens available in collections refer to the following collections: BEV.[X]: Laboratoire de Biogéographie et Écologie des Vertébrés de l'École Pratique des Hautes Etudes, Montpellier, France; NHMC.[X]: Natural History Museum of Crete, Greece; MCC[X]: Museo Civico di Storia Naturale di Carmagnola, Italy; HUJR-[X]: National Natural History Collections of the Hebrew University of Jerusalem, Israel; CUP[X]: Charles University, Prague; JFBM[X]: James Ford Bell Museum, Amphibian and Reptile Collection, University of Minnesota. Codes BEV.T[X] refer to the tissue collection of Laboratoire de Biogéographie et Écologie des Vertébrés de l'École Pratique des Hautes Etudes, Montpellier, France.
