# Supplementary figures and images for: Conquering the Sahara and Arabian deserts: systematics and biogeography of Stenodactylus geckos (Reptilia: Gekkonidae)
Source: BMC Evol Biol. 2012 Dec 31;12:258. doi: 10.1186/1471-2148-12-258 (PMC3582542; doi:10.1186/1471-2148-12-258)

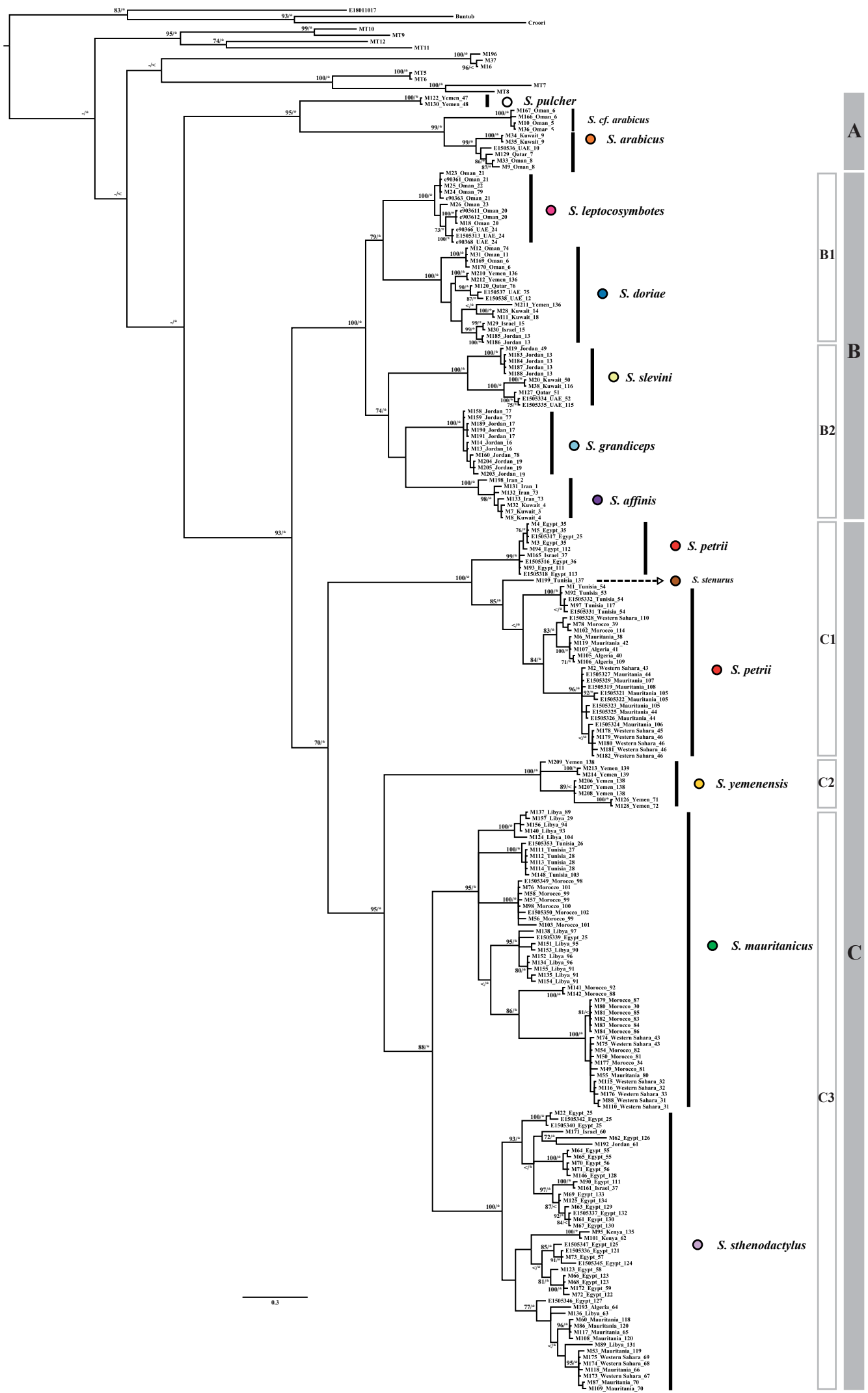

Supplement: Additional file 2: Figure S1 — BI tree of the genus Stenodactylus inferred using 12S and 16S mtDNA gene fragments. Description of data: Posterior probability values above 0.95 in the Bayesian Inference analysis are indicated next to the nodes with an asterisk, while numbers correspond to bootstrap support of the Maximum Likelihood analysis (only values above 70 are shown). The tree was rooted using Hemidactylus frenatus. Numbers in square brackets next to specimen code refer to Figure 1. Information on the samples included is shown in Additional file 1: Table S1. [file 1471-2148-12-258-S2.pdf]

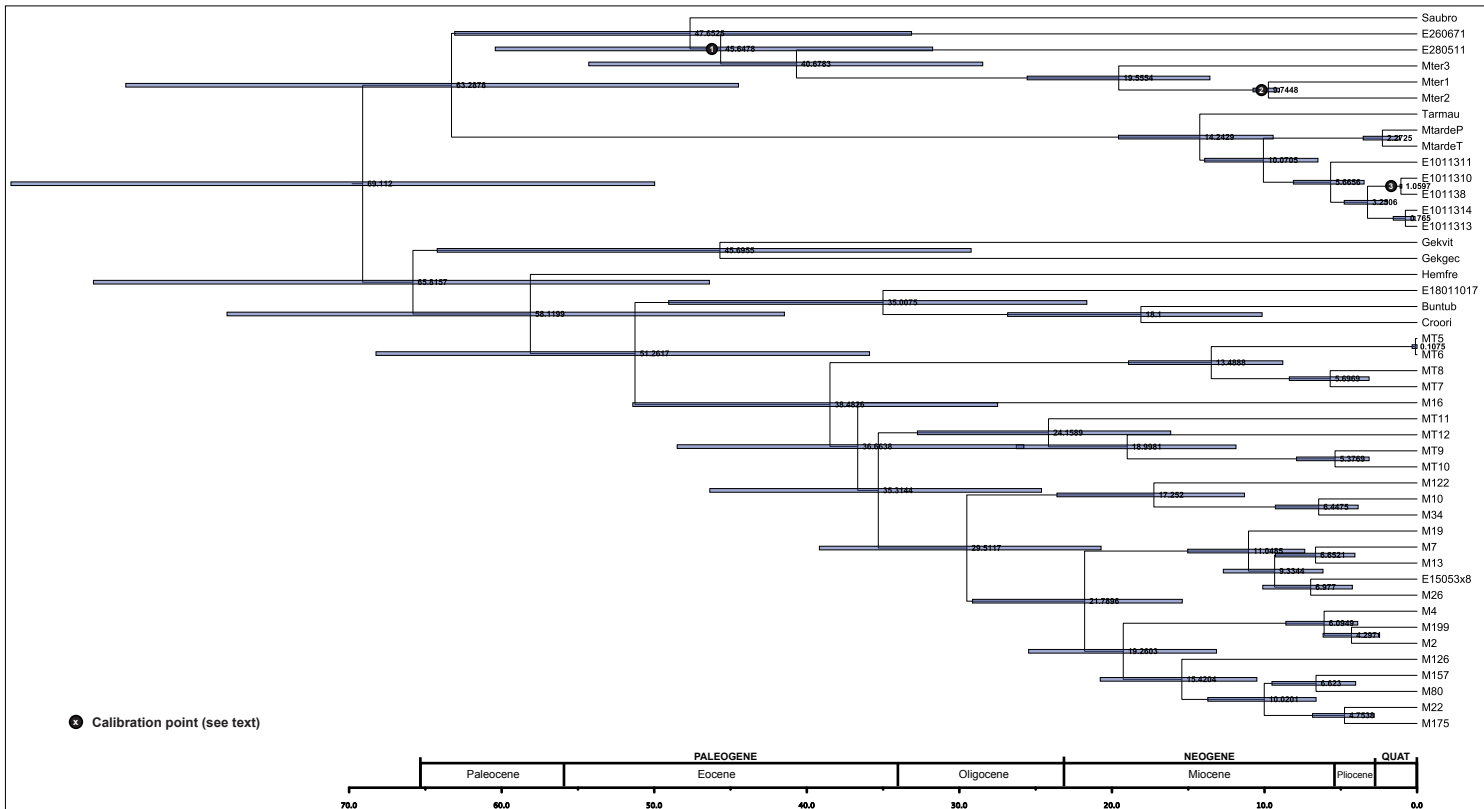

Supplement: Additional file 4: Figure S2 — Chronogram obtained with BEAST inferred using all markers and 3 calibration points. Description of data: Chronogram obtained with relaxed uncorrelated lognormal clock and Yule model of speciation. Filled numbered circles correspond to calibration points described in Materials and Methods. The x axis is in million years and the bars indicate 95% HPD intervals. Information on the samples included is shown in Additional file 1: Figure S1. [file 1471-2148-12-258-S4.pdf]
