# Supplementary material for: Conquering the Sahara and Arabian deserts: systematics and biogeography of Stenodactylus geckos (Reptilia: Gekkonidae)
Source: BMC Evol Biol. 2012 Dec 31;12:258. doi: 10.1186/1471-2148-12-258 (PMC3582542; doi:10.1186/1471-2148-12-258)
Supplement: Additional file 3: Table S2 — Uncorrected p-distances (pairwise deletion). [file 1471-2148-12-258-S3.doc]

**Table S2** **Uncorrected** ***p*-distances (pairwise deletion)**

| **a. Between species** | | | | | | | | | | | | | | | | | | | |
| --- | --- | --- | --- | --- | --- | --- | --- | --- | --- | --- | --- | --- | --- | --- | --- | --- | --- | --- | --- |
|  | **Talg** | **Ttrip** | **Tnatt** | **Tscort** | **Tsteud** | **Tnub** | **khob** | **pulch** | **arab** | **lepto** | **dori** | **slev** | **grandi** | **affi** | **petr** | **stenu** | **yeme** | **mauri** | **stheno** |
| Talg |  | 0.082 | 0.155 | n/c | 0.164 | 0.158 | 0.148 | 0.169 | 0.153 | 0.157 | 0.162 | 0.163 | 0.166 | 0.166 | 0.166 | 0.170 | 0.187 | 0.164 | 0.158 |
| Ttrip | 0.120 |  | 0.173 | n/c | 0.186 | 0.184 | 0.161 | 0.168 | 0.165 | 0.167 | 0.161 | 0.179 | 0.160 | 0.172 | 0.175 | 0.180 | 0.187 | 0.172 | 0.169 |
| Tnatt | 0.172 | 0.194 |  | n/c | 0.143 | 0.133 | 0.169 | 0.173 | 0.161 | 0.139 | 0.147 | 0.170 | 0.144 | 0.162 | 0.151 | 0.152 | 0.177 | 0.160 | 0.154 |
| Tscort | 0.199 | 0.199 | 0.159 |  | n/c | n/c | n/c | n/c | n/c | n/c | n/c | n/c | n/c | n/c | n/c | n/c | n/c | n/c | n/c |
| Tsteud | 0.161 | 0.181 | 0.151 | 0.125 |  | 0.057 | 0.180 | 0.194 | 0.189 | 0.167 | 0.174 | 0.175 | 0.177 | 0.183 | 0.167 | 0.170 | 0.179 | 0.164 | 0.163 |
| Tnub | 0.154 | 0.173 | 0.159 | 0.151 | 0.057 |  | 0.167 | 0.185 | 0.185 | 0.156 | 0.163 | 0.168 | 0.170 | 0.176 | 0.166 | 0.165 | 0.180 | 0.160 | 0.158 |
| khob | 0.190 | 0.192 | 0.214 | 0.199 | 0.187 | 0.173 |  | 0.168 | 0.152 | 0.146 | 0.166 | 0.159 | 0.158 | 0.164 | 0.175 | 0.174 | 0.178 | 0.149 | 0.147 |
| pulch | 0.181 | 0.201 | 0.192 | 0.190 | 0.166 | 0.161 | 0.206 |  | 0.145 | 0.156 | 0.155 | 0.148 | 0.162 | 0.168 | 0.166 | 0.182 | 0.176 | 0.140 | 0.140 |
| arab | 0.206 | 0.219 | 0.220 | 0.216 | 0.189 | 0.206 | 0.222 | 0.125 |  | 0.157 | 0.165 | 0.159 | 0.157 | 0.163 | 0.177 | 0.173 | 0.179 | 0.152 | 0.141 |
| lepto | 0.190 | 0.217 | 0.189 | 0.201 | 0.182 | 0.185 | 0.230 | 0.182 | 0.206 |  | 0.068 | 0.079 | 0.065 | 0.074 | 0.111 | 0.115 | 0.128 | 0.119 | 0.110 |
| dori | 0.189 | 0.226 | 0.186 | 0.197 | 0.179 | 0.190 | 0.236 | 0.186 | 0.194 | 0.063 |  | 0.083 | 0.064 | 0.076 | 0.116 | 0.117 | 0.133 | 0.117 | 0.114 |
| slev | 0.195 | 0.204 | 0.205 | 0.208 | 0.192 | 0.200 | 0.218 | 0.196 | 0.214 | 0.094 | 0.099 |  | 0.092 | 0.092 | 0.122 | 0.134 | 0.139 | 0.126 | 0.112 |
| grandi | 0.199 | 0.213 | 0.195 | 0.203 | 0.186 | 0.192 | 0.225 | 0.194 | 0.208 | 0.070 | 0.094 | 0.076 |  | 0.072 | 0.113 | 0.114 | 0.120 | 0.104 | 0.102 |
| affi | 0.202 | 0.232 | 0.219 | 0.218 | 0.205 | 0.213 | 0.242 | 0.209 | 0.211 | 0.076 | 0.097 | 0.093 | 0.068 |  | 0.130 | 0.131 | 0.133 | 0.118 | 0.109 |
| petr | 0.218 | 0.226 | 0.227 | 0.220 | 0.200 | 0.195 | 0.235 | 0.178 | 0.217 | 0.148 | 0.167 | 0.183 | 0.163 | 0.169 |  | 0.045 | 0.122 | 0.111 | 0.119 |
| stenu | 0.206 | 0.217 | 0.224 | 0.210 | 0.199 | 0.197 | 0.216 | 0.183 | 0.209 | 0.142 | 0.161 | 0.174 | 0.156 | 0.162 | 0.056 |  | 0.130 | 0.116 | 0.118 |
| yeme | 0.193 | 0.217 | 0.212 | 0.205 | 0.192 | 0.195 | 0.209 | 0.208 | 0.231 | 0.190 | 0.191 | 0.193 | 0.187 | 0.201 | 0.175 | 0.167 |  | 0.114 | 0.099 |
| mauri | 0.196 | 0.226 | 0.209 | 0.218 | 0.196 | 0.197 | 0.237 | 0.183 | 0.189 | 0.148 | 0.158 | 0.177 | 0.159 | 0.160 | 0.166 | 0.162 | 0.156 |  | 0.072 |
| stheno | 0.195 | 0.215 | 0.213 | 0.234 | 0.196 | 0.195 | 0.247 | 0.194 | 0.207 | 0.165 | 0.179 | 0.189 | 0.169 | 0.182 | 0.187 | 0.182 | 0.158 | 0.109 |  |
| **b. Within species** | | | | | | | | | | | | | | | | | | | |
|  | khob | pulch | arab | lepto | dori | slev | grandi | affi | petr | stenu | yeme | mauri | stheno |  |  |  |  |  |  |
| 12S | 0.005 | 0.000 | 0.045 | 0.009 | 0.029 | 0.030 | 0.002 | 0.029 | 0.044 | n/c | 0.060 | 0.047 | 0.047 |  |  |  |  |  |  |
| 16S | 0.007 | 0.000 | 0.033 | 0.004 | 0.022 | 0.022 | 0.002 | 0.008 | 0.038 | n/c | 0.009 | 0.043 | 0.032 |  |  |  |  |  |  |
| **c. Between intraspecific groups** | | | | | | | | | | | | | | | | | | | |
|  | arab_EOma | arab_Arabia | dori_EOma | dori_Arabia | petr_Egy | petr | stheno_NEgy | stheno_SEgy | stheno_West | mauri_ELib | mauri_WLib | mauri_Tun | mauri_NMor | mauri_CMor | mauri_WSah |  |  |  |  |
| arab_cf |  | 0.050 | 0.156 | 0.155 | 0.164 | 0.175 | 0.135 | 0.130 | 0.137 | 0.147 | 0.156 | 0.151 | 0.151 | 0.157 | 0.142 |  |  |  |  |
| arab_Arabia | 0.077 |  | 0.173 | 0.172 | 0.174 | 0.184 | 0.146 | 0.141 | 0.147 | 0.150 | 0.165 | 0.156 | 0.152 | 0.150 | 0.154 |  |  |  |  |
| dori_EOma | 0.184 | 0.197 |  | 0.027 | 0.114 | 0.125 | 0.113 | 0.113 | 0.112 | 0.100 | 0.115 | 0.118 | 0.116 | 0.114 | 0.123 |  |  |  |  |
| dori_Arabia | 0.189 | 0.200 | 0.040 |  | 0.111 | 0.116 | 0.116 | 0.114 | 0.114 | 0.103 | 0.115 | 0.122 | 0.119 | 0.116 | 0.123 |  |  |  |  |
| petr_Egy | 0.223 | 0.226 | 0.173 | 0.178 |  | 0.060 | 0.111 | 0.106 | 0.110 | 0.108 | 0.109 | 0.117 | 0.121 | 0.104 | 0.099 |  |  |  |  |
| petr | 0.208 | 0.219 | 0.161 | 0.165 | 0.072 |  | 0.125 | 0.120 | 0.124 | 0.115 | 0.115 | 0.119 | 0.130 | 0.114 | 0.107 |  |  |  |  |
| stheno_NEgy | 0.198 | 0.207 | 0.175 | 0.175 | 0.192 | 0.186 |  | 0.039 | 0.042 | 0.062 | 0.079 | 0.068 | 0.069 | 0.081 | 0.078 |  |  |  |  |
| stheno_SEgy | 0.195 | 0.206 | 0.177 | 0.180 | 0.188 | 0.185 | 0.058 |  | 0.032 | 0.060 | 0.071 | 0.064 | 0.066 | 0.075 | 0.068 |  |  |  |  |
| stheno_West | 0.210 | 0.218 | 0.186 | 0.185 | 0.187 | 0.189 | 0.059 | 0.051 |  | 0.067 | 0.081 | 0.071 | 0.073 | 0.084 | 0.077 |  |  |  |  |
| mauri_ELib | 0.174 | 0.188 | 0.145 | 0.154 | 0.164 | 0.161 | 0.099 | 0.100 | 0.111 |  | 0.043 | 0.049 | 0.041 | 0.043 | 0.052 |  |  |  |  |
| mauri_WLib | 0.180 | 0.202 | 0.136 | 0.148 | 0.163 | 0.159 | 0.106 | 0.110 | 0.116 | 0.045 |  | 0.056 | 0.046 | 0.060 | 0.064 |  |  |  |  |
| mauri_Tun | 0.178 | 0.195 | 0.134 | 0.143 | 0.158 | 0.158 | 0.096 | 0.103 | 0.112 | 0.040 | 0.049 |  | 0.042 | 0.060 | 0.063 |  |  |  |  |
| mauri_NMor | 0.198 | 0.199 | 0.154 | 0.162 | 0.169 | 0.162 | 0.112 | 0.104 | 0.120 | 0.046 | 0.049 | 0.047 |  | 0.060 | 0.066 |  |  |  |  |
| mauri_CMor | 0.160 | 0.187 | 0.138 | 0.146 | 0.170 | 0.161 | 0.096 | 0.094 | 0.109 | 0.046 | 0.052 | 0.049 | 0.058 |  | 0.048 |  |  |  |  |
| mauri_WSah | 0.178 | 0.194 | 0.166 | 0.172 | 0.171 | 0.174 | 0.111 | 0.112 | 0.118 | 0.061 | 0.066 | 0.070 | 0.074 | 0.052 |  |  |  |  |  |

12S: lower left, 16S: upper right. Abbreviations: Talg: *Tropiocolotes algirus*, Ttrip: *T. tripolitanus*, Tnatt: *T. nattereri*, Tscort: *T. scortecci*, Tnub: *T. nubicus*, khob: *Pseudoceramodactylus khobarensis*, pulch: *Stenodactylus pulcher*, arab: *S. arabicus*, lepto: *S. leptocosymbotes*, dori: *S. doriae*, slev: *S. slevini*, grandi: *S. grandiceps*, affi: *S. affinis*, petr: *S. petrii*, stenu: *S. stenurus*, mauri: *S. mauritanicus*, stheno: *S. sthenodactylus.*
